# Heat and mass transfer analysis for magnetized flow of ${\mathrm{ZnO}-SAE50}$ nanolubricant with variable properties: an application of Cattaneo–Christov model

**DOI:** 10.1038/s41598-023-35988-7

**Published:** 2023-05-30

**Authors:** Muhammad Riaz, Nargis Khan, M. S. Hashmi, Jihad Younis

**Affiliations:** 1 Department of Mathematics, The Islamia University of Bahawalpur, Bahawalpur, Pakistan; 2 Department of Mathematics, The Govt. Sadiq College Women University, Bahawalpur, Pakistan; 3 Department of Mathematics, Aden University, P.O. Box 6014, Aden, Yemen

**Keywords:** Nanoscience and technology, Applied mathematics

## Abstract

The current study scrutinizes heat and mass transfer features of magnetized flow of ${\mathrm{ZnO}-SAE50}$ nanolubricant over Riga plate in a Darcy Forchheimer medium. The effects of variable viscosity, thermal radiation, variable thermal conductivity, viscous dissipation and uniform heat source/sink are examined in this study. The diffusion model presented by Cattaneo–Christov is incorporated in this study to enclose heat and mass transport phenomenon. Additionally, the mass transfer rate is inspected subjected to the effects of variable solutal diffusivity and higher order chemical reaction. Heat and mass transfer phenomena have significant applications in the disciplines of science and technology that can be seen everywhere in nature. This simultaneous transportation phenomenon indicates a variety of applications in manufacturing processes, aerodynamics, cooling systems, environmental sciences, oceanography, food industries, biological disciplines, and energy transport systems etc. The modeled system of PDEs is metamorphosed to nonlinear ODEs with the introduction of appropriate transformations. An eminent bvp4c method in MATLAB has been incorporated to execute the resulting system of ODEs numerically. The outcomes of velocity, temperature and concentration profiles corresponding to various emerging parameters have been exposed graphically. The motion of ${\mathrm{ZnO}-SAE50}$ nanolubricant tends to enhance significantly with larger modified Hartmann number, whereas converse behavior is reported by increasing porosity parameter and variable viscosity parameter. The greater heat transfer rate is observed for variable thermal conductivity parameter. The rates of heat and mass transfer slow down for thermal and solutal time relaxation parameters respectively. The concentration profile gets enriched by growing the order of the chemical reaction and variable mass diffusivity parameter. It is concluded that by increasing solid volume fraction up to $1.5\%$, the viscosity of the nanolubricant enhances up to $12\%$ which consequently slows down motion of the nanolubricant but increases temperature and concentration profiles.

## Introduction

In recent years, one of the main concerns for scientists and engineers is managing the flow of electrically conducting fluids. In industrial and technological operations, such as those involving mass and heat transfer, these fluids can be flown under controlled conditions in a variety of ways. However, with the help of electromagnetic body forces in the polymer sector, researchers have introduced few traditional methods for controlling fluid flow, including blowing/suction and wall motion methods. Applying an external magnetic field can drastically alter the fluids’ flow with greater electrical conductivity, such as liquid metals, electrolytes and plasma etc. The magnetic field plays a significant role in fluid mechanics due to its multiple uses in strengthening a fluid's thermo-physics properties. Prasannakumara and Gowda^1^ investigated heat and mass transfer features of radiative flow under the effects of thermophoretic particle deposition and uniform magnetic field. Umavathi et al.^2^ discussed squeezing flow of magnetized Casson nanofluid between parallel disks. In disciplines like earth sciences and astrology, several poor electrically conductive liquids are encountered. In order to boost heat flow rate through enhanced conductivity and other thermo-physical qualities, an exterior agent is frequently needed. This exterior agent can be a magnetic component or a permanently fixed series of magnets with discontinuous electrodes. The Riga plate was formally introduced by Gailitis^3^, who was the first to employ this kind of formulation. Since it has acquired widespread acceptance in industrial processes that influence the behaviour of fluid flow, the Riga plate is particularly advantageous in its current configuration. Shafiq et al.^4^ used Walters-B model to study fluid flow over a Riga plate. To examine nanoparticles behavior and mixed convection in a fluid flow, Adeel et al.^5^ used a Riga plate that was positioned vertically. Rasool et al.^6^ looked into how thermal radiation affects the nanoliquid flow over a Riga plate.

The distinct physical and chemical properties of suspended nanoparticles are piquing the curiosity of engineers and scientists. The thermal performance of these nanometer-sized particles is predicted to be greater than that of ordinary fluids. Nanofluids are essentially basic fluids with nanoparticles dispersed in them. Numerous factors, including Brownian motion, chain creation of nanoparticles, crystallization at the solid–fluid interface, and friction between fluid and particles, affect the impact of nanofluid. The term "nanofluid," which states that the rate of heat transmission can be greatly boosted by increasing the thermal potential of conventional fluid, was firstly coined by Choi^7^. Alsulami et al.^8^ used Buongiorno model to analyze bioconvection in radiative Glauert wall jet flow of nanofluid. The nanoparticles are frequently divided into six different types including carbon nanoparticles, nano-composites, metals, sulphides, metal oxides and rare earth metals^9^, while the base liquid is typically an electrically conductive liquid like oil, water or ethylene glycol. The solid metals have greater thermal conductivity in comparison to base fluids. As a result, thermal conductivity and heat transfer performance can be boosted by suspended nanoparticles. Hamid et al.^10^ numerically analyzed the magneto-cross nanofluid’s flow with effective Prandtl number approach in the presence of gyrotactic microorganisms. Gowda et al.^11^ investigated the dynamics of interfacial layer and nanoparticle diameter on non-Newtonian (Jeffrey) nanofluid flow. The studies^12–19^ investigate the behavior of some nanofluids comprising different nanoparticles. The most prevalent type of nanoparticle found in petroleum oils is metal oxide. About $26\%$ of the nanoparticles utilized in lubricating oils are metal oxides^9^. In present work, ${\mathrm{ZnO}-SAE50}$ nanolubricant which is the mixture of metal oxide nanoparticles ${\mathrm{ZnO}}$ in base lubricant $SAE50$, has been considered for the analysis. The imperative to fulfill diverse industrial demands drove the development of ${\mathrm{ZnO}-SAE50}$ nanolubricant, a cutting-edge solution that significantly enhances heat transfer rates, making it an indispensable asset for industries striving to optimize their performance.

The phenomenon of heat transmission is essential in industrial, natural, geophysical and biotechnological systems. Heat transfer is a phenomenon brought on by temperature changes between objects or between various areas of the same object. In engineering and biological sectors, this phenomenon has plenty of applications such as energy production, drying technology, catalytic reactors, cooling of atomic reactor, medication and heat conveyance in tissues etc. Kumar et al.^20^ conducted a study to inspect convective heat transfer and KKL correlation to simulate flow of nanofluid over a stretching sheet. Alsulami et al.^21^ investigated heat transmission rate in a porous medium for a non‐Newtonian fluid comprising $AA7075$ and $Ti6Al4V$ nanoparticles using Local thermal non-equilibrium conditions. Kumar et al.^22^ presented a comparative heat transfer analysis in magnetized flow of ternary hybrid nanofluid incorporated with three various shaped nanoparticles. In order to better understand the heat transfer phenomenon, Fourier^23^ was the pioneer to propose a heat transmission mechanism. However, this model had to be updated by numerous researchers due to various drawbacks, such as its inability to produce parabolic energy transport equation for temperature fields. One of them that has been successful is the Cattaneo^24^ model, which has thermal relaxation time to represent thermal inertia. After taking into account relaxation period, hyperbolic kind energy equation becomes apparent, allowing for the limited-speed propagation of thermal waves to transfer heat. Later, Christov^25^ changed the time derivative to an Oldroyd derivative. The Cattaneo–Christov model is the name given to it after this modification. This updated version has been applied in numerous recent investigations^26–29^. Sarada et al.^30^ used non-Fourier heat flux model to discuss the effect of exponential form of internal heat generation on the flow ternary hybrid nanofluid.

A material's ability to conduct heat is referred to as its thermal conductivity. It is a material’s property that is supposed to vary with temperature. Thermal conductivity in fluids is based on by two distinct processes. One, as the rate of molecule collisions increases, so do the rates of energy exchange, which helps to move heat across the medium. Second, the presence of thermal conductivity upsurges the amount of molecules’ random movement. When the amount of molecules random movement rises, heat energy increases. It is fairly obvious that poor conductive materials are utilized in insulation and materials with greater thermal conductivity are employed in heat sinks. Applications of thermal conductivity include steam generators, electrolytes, heating concrete, catalysis, laminating, and molding blow. The impact of variable thermal conductivity on the flow of nanofluid over a moving thin needle was investigated by Khan et al.^31^. Williamson nanofluid flow across a stretching surface with variable thermal conductivity was taken into account by Reddy et al.^32^. Variable thermal conductivity with various geometries has recently been studied by certain researchers^33–36^.

The amount of fluid’s resistance to flow is known as its viscosity. The use of viscosity is crucial to many technological and industrial practices. The fluid flow in heavy extrusion processes, thermal dynamic systems, and the transport phenomenon change when thermal conductivity and viscosity of the material vary as a result of diverse substances. Typically, variations in temperature, pressure and shear rate cause changes in fluid viscosity. Using radiated material as an example, Aldabesh et al.^37^ described the variable nature of viscosity. The variable viscosity was used by Olabode et al.^38^ to describe the essential elements of heat transportation in porous space. The influence of thermal radiation and temperature-dependent viscosity on slip flow over a convicting sheet was addressed by Iqbal et al.^39^.

In the above mentioned literature, majority of the studies on heat and mass transfer assessment of nanofluids in view of Cattaneo–Christov heat and mass flux model under various aspects have been conducted by taking water or other base fluids. Only limited studies exist in the literature that considered lubricating oils rather than water and other base fluids. The present study primarily investigates the flow of chemically reactive magnetized ${\mathrm{ZnO}-SAE50}$ nanolubricant over Riga plate in Darcy Forchheimer media with viscous dissipation, heat sink/source, thermal radiation and Cattaneo–Christove heat and mass flux model. The role of viscosity, thermal conductivity and solutal diffusivity is presumed to be variable to make the study more convenient. To the best of our expertise, this investigation (including these aspects) is a new discovery with plenty of applications in mechanics and fills the gap not satisfactorily spoken in the prevailing literature by answering the following key questions:

- What effects do the magnetic parameter and modified Hartmann number have on the velocity of the nanolubricant ${\mathrm{ZnO}-SAE50}$?
- How does the velocity of the nanolubricant ${\mathrm{ZnO}-SAE50}$ get affected in the Darcy Forchheimer medium?
- What influence does the variable viscosity parameter have on the motion of the nanolubricant ${\mathrm{ZnO}-SAE50}$?
- How do the thermal radiation and heat source-sink parameters alter the temperature of the nanolubricant ${\mathrm{ZnO}-SAE50}$?
- How do the heat and mass transmission phenomena are affected with variable thermal conductivity and variable diffusivity parameters?
- What impacts do the solutal and thermal relaxation parameters impart on temperature and concentration of the nanolubricant?
- How does the enhancing order of the chemical reaction affect the concentration profile?
- What effect does the solid volume fraction parameter have on the velocity, temperature and concentration profiles?

## Mathematical development

**Figure 1:**
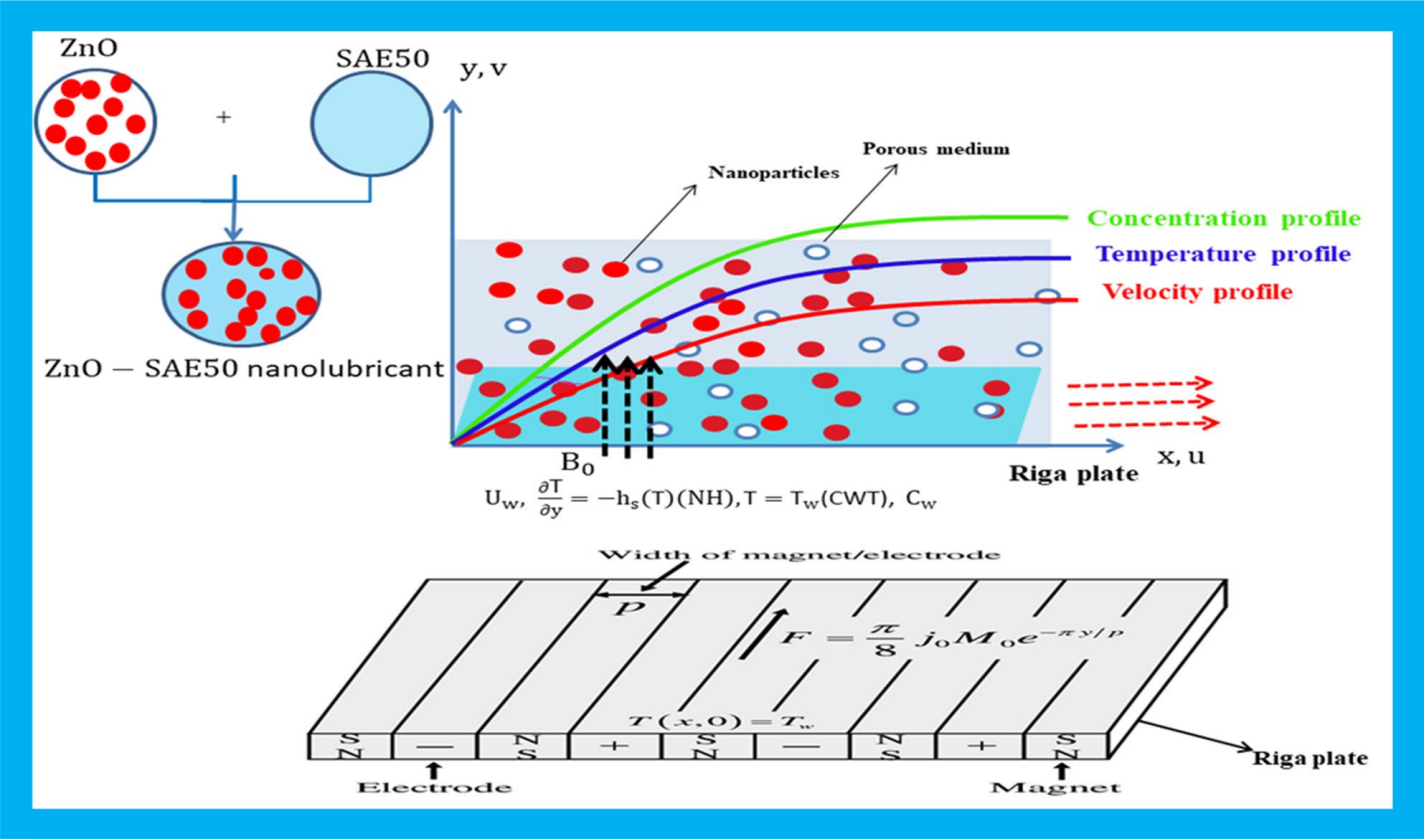
Physical model of the flow.

This segment merely concerns with the evidence regarding governing equations’ formulation under some assumptions. Here, we have considered a steady, laminar and incompressible flow of ${\mathrm{ZnO}-SAE50}$ nanolubricant over a Riga plate as presented in Fig. 1. The Riga plate is supposed under electromagnetic force ${F}_{m}=\left({F}_{m},\mathrm{0,0},\right)$, $\left({F}_{m}=\frac{\pi {j}_{0}{M}_{0}}{\delta {\rho }_{nf}}\mathit{exp}\left(-\frac{\pi }{a}y\right)\right).$ The consecutive arrays of permanent electrodes and magnets organized on a smooth surface set up a Riga plate. The Riga plate is presumed to be moving with uniform velocity ${U}_{w}=cx$ in the $x-$ direction, and $y-$ axis is vertical to the motion of the nanolubricant. At the Riga plate surface (at $y=0$), concentration and temperature are set as ${C}_{w}$ and ${T}_{w}$, and away from the surface (as $y\to \infty$) they are represented as ${C}_{\infty }$ and ${T}_{\infty }\left({T}_{\infty }<{ T}_{w}\right)$ respectively.

### Flow assumptions

The analysis is carried out under the following assumptions:

- The flow of nanolubricant $({\mathrm{ZnO}-SAE50})$ is considered.
- Darcy Forchheimer medium is considered into account.
- A magnetic field having strength ${B}_{0}$ is employed.
- The role of viscosity, thermal conductivity and solutal diffusivity is presumed to be variable.
- The impacts of nonlinear heat radiation are deliberated.
- Uniform heat sink/source is applied.
- Viscous dissipation is included in the modeling.
- Cattaneo–Christov model is applied.
- Newtonian heating condition is implemented.
- Mass transfer rate is further inspected by applying higher order chemical reaction.

The equations that govern the flow of the nanolubricant ${\mathrm{ZnO}-SAE50}$ under the aforementioned assumptions are specified as^40,41^

#### Continuity equation

1 $$\frac{\partial u}{\partial x}+\frac{\partial v}{\partial y}=0$$

#### Momentum equation

2 $$u\frac{\partial u}{\partial x}+v\frac{\partial u}{\partial y}=\frac{1}{{\rho }_{\mathrm{ZnO}-SAE50 \, }}\frac{\partial }{\partial y}\left({\mu }_{\mathrm{ZnO}-SAE50 \, }^{*}\left(T\right)\frac{\partial u}{\partial y}\right)+\frac{\pi {j}_{0}{M}_{0}}{8{\rho }_{nf}}\mathit{exp}\left(-\frac{\pi }{p}y\right)-\frac{{{\mu }^{*}}_{\mathrm{ZnO}-SAE50 \, }\left(T\right)}{{\rho }_{\mathrm{ZnO}-SAE50 \, }}\frac{u}{{k}^{*}} -\frac{{\sigma }_{\mathrm{ZnO}-SAE50 \, }}{{\rho }_{\mathrm{ZnO}-SAE50 \, }}{B}_{0}^{2}u-F{u}^{2}$$

#### Energy equation

3 $$u\frac{\partial T}{\partial x}+v\frac{\partial T}{\partial y}= \frac{1}{{\left(\rho {C}_{p}\right)}_{\mathrm{ZnO}-SAE50}}\frac{\partial }{\partial y}\left({k}_{\mathrm{ZnO}-SAE50}^{*}\left(T\right)\frac{\partial T}{\partial y}\right)-{\lambda }_{H}{T}_{H}+\frac{{Q}_{0}}{{\left(\rho {C}_{p}\right)}_{\mathrm{ZnO}-SAE50}}\left(T-{T}_{\infty }\right)+\frac{1}{{\left(\rho {C}_{p}\right)}_{\mathrm{ZnO}-SAE50}}\frac{\partial {q}_{r}}{\partial \mathrm{y}}+\frac{{{\mu }^{*}}_{\mathrm{ZnO}-SAE50 \, }\left(T\right)}{{\left(\rho {C}_{p}\right)}_{\mathrm{ZnO}-SAE50}}{\left(\frac{\partial u}{\partial y}\right)}^{2}$$

#### Concentration equation

4 $$u\frac{\partial C}{{\partial x}} + v\frac{\partial C}{{\partial y}} = \frac{\partial }{\partial y}\left( {D_{{{\text{ZnO}} - SAE50}}^{*} \left( {\text{C}} \right)\frac{\partial C}{{\partial y}}} \right) - \lambda_{M} C_{M} - k_{0} \left( {{\text{C}} - C_{\infty } } \right)^{m}$$

where ${T}_{H}$ and ${C}_{M}$ are given as

5 $${T}_{H}=\left({u}^{2}\frac{{\partial }^{2}T}{\partial {x}^{2}}+u\frac{\partial u}{\partial x}\frac{\partial T}{\partial x}+u\frac{\partial v}{\partial x}\frac{\partial T}{\partial x}+2uv\frac{{\partial }^{2}T}{\partial x\partial y}+v\frac{\partial v}{\partial y}\frac{\partial T}{\partial y}+v\frac{\partial u}{\partial y}\frac{\partial T}{\partial x}+{v}^{2}\frac{{\partial }^{2}T}{\partial {y}^{2}}\right)$$

6 $${C}_{M}=\left({u}^{2}\frac{{\partial }^{2}C}{\partial {x}^{2}}+u\frac{\partial u}{\partial x}\frac{\partial C}{\partial x}+u\frac{\partial u}{\partial x}\frac{\partial C}{\partial y}+2uv\frac{{\partial }^{2}C}{\partial x\partial y}+v\frac{\partial v}{\partial y}\frac{\partial C}{\partial y}+v\frac{\partial u}{\partial y}\frac{\partial C}{\partial x}+{v}^{2}\frac{{\partial }^{2}C}{\partial {y}^{2}}\right)$$

Equation (1) represents the equation of continuity, whereas Eqs. (2, 3, 4) represent equations of momentum, energy and concentration respectively. Eqs. (5) and (6) represent heat and mass fluxes correspondingly. The BCs are set as^42,43^.

7 $$u={U}_{w}=cx, v=0, T={T}_{w}\left(CWT\right), \frac{\partial T}{\partial y}=-{h}_{s}T\left(NH\right), C={C}_{w}\; \mathrm{at }\;y=0$$

8 $$u=0, T\to { T}_{\infty }, C\to {C}_{\infty } \;as\; y\to \infty$$

### Cattaneo–Christov diffusion model

The concentration and thermal diffusion are characterized by the Cattaneo–Christov diffusion model, which is introduced with the relaxation of the mass and heat flux. The generalization of Fick's and Fourier's law known as the Cattaneo–Christov double diffusion model is then derived as

9 $$J+{\lambda }_{C}\left[\frac{\partial J}{\partial t}+V\cdot \nabla J-J\cdot \nabla V+(\nabla \cdot V)J\right]=-{D}_{B}\nabla C$$

10 $$q+{\lambda }_{E}\left[\frac{\partial q}{\partial t}+V\cdot \nabla q-q\cdot \nabla V+(\nabla \cdot V)q\right]=-{k}_{f}\nabla T$$

where, $J-$ normal mass flux, ${k}_{f}-$ thermal conductivity,${D}_{B}-$ Brownian diffusion coefficient,${\lambda }_{E}-$ relaxation time of heat flux , $q-$ normal heat flux and ${\lambda }_{C}-$ relaxation time mass flux. Eqs. (9) and (10) are reduced to the classical Fick's law and Fourier's law when ${\lambda }_{C}=0$ and ${\lambda }_{E}=0$. Eqs. (9) and (10) can be written as follows when the continuity equation $\nabla \cdot V=0$ and the constant laminar flow $\partial q/\partial t=0$ and $\partial J/\partial t=0$ are taken into account.

11 $$J+{\lambda }_{C}\left[V\cdot \nabla J-J\cdot \nabla V\right]=-{D}_{B}\nabla C.$$

12 $$q+{\lambda }_{E}\left[V\cdot \nabla q-q\cdot \nabla V\right]=-{k}_{f}\nabla T$$

### Variable viscosity

The relation for variable viscosity (temperature dependent) is presented as^44^.

13 $${{\mu }_{\mathrm{ZnO}-SAE50 \, }^{*}\left(T\right)=\mu }_{{\mathrm{ZnO}-SAE50} }{e}^{-{\epsilon }_{1}\left(\frac{T-{T}_{\infty }}{{T}_{w}-{T}_{\infty }}\right)}$$

Generally, ${\epsilon }_{1}>0$ for liquids and ${\epsilon }_{1}<0$  for gases.

### Variable thermal conductivity

The temperature-dependent thermal conductivity can be defined as^45^

14 $${k}_{\mathrm{ZnO}-SAE50 \, }^{*}\left(T\right)={k}_{\mathrm{ZnO}-SAE50}\left[1+{\epsilon }_{2}\left(\frac{T-{T}_{\infty }}{{T}_{w}-{T}_{\infty }}\right)\right]$$

### Variable concentration relation

The variable diffusivity can be defined as^46^

15 $${D}_{\mathrm{ZnO}-SAE50 \, }^{*}\left(C\right)={D}_{\mathrm{ZnO}-SAE50}\left[1+{\epsilon }_{3}\left(\frac{C-{C}_{\infty }}{{C}_{w}-{C}_{\infty }}\right)\right]$$

### Rosseland approximation

The radiative heat flow term can be simplified by utilizing Rosseland approximation and is defined as^47^.

16 $${q}_{r}=-\frac{4{\sigma }^{*}}{3{k}^{*}}\frac{\partial {T}^{4}}{\partial y}$$

where ${\sigma }^{*}-$ Stefan–Boltzman constant and ${k}^{*}-$ absorption coefficient. We can express the term ${T}^{4}$ as a linear function of temperature if the temperature variations within the fluid in the boundary layer are sufficiently small. Thus, by neglecting higher-order terms and expanding ${T}^{4}$ in a Taylor series around ${T}_{\infty }$, we can obtain ${T}^{4}\approx 4T{T}_{\infty }^{3}-3{T}_{\infty }^{4}$. Now, Eq. (16) can be represented as

17 $${q}_{r}=-\frac{16{\sigma }^{*}{T}_{\infty }^{3}}{3{k}^{*}}\frac{\partial T}{\partial y}$$

### Thermo physical properties of $\mathbf{Z}\mathbf{n}\mathbf{O}-{\varvec{S}}{\varvec{A}}{\varvec{E}}50$ nano-lubricant

The heat capacitance, effective density and viscosity of $\mathrm{ZnO}-SAE50$ nano-lubricant are^48,49^

18 $${\left(\rho {C}_{p}\right)}_{\mathrm{ZnO}-SAE50}=(1-\phi ){\left(\rho {C}_{p}\right)}_{SAE50}+\phi {\left(\rho {C}_{p}\right)}_{\mathrm{ZnO}}$$

19 $${\rho }_{\mathrm{Zn}O-SAE50}=(1-\phi ){\rho }_{\text{SAE50 }}+\phi {\rho }_{\mathrm{Zn}O}$$

20 $${\mu }_{\mathrm{ZnO}-SAE50}={\mu }_{\text{SAE50 }}(1.035+0.04336\phi -0.002407T)$$

21 $${\mu }_{\text{SAE50 }}=2.174{e}^{-0.06062T}$$

where, $\phi$ and $T$ symbolize volume fraction $(\%)$ and temperature (degree centigrade).

Patel et al.^50^ proposed the following relation to predict effective thermal conductivity of nano-lubricant

22 $$\left.\begin{array}{c}\frac{{k}_{\mathrm{ZnO}-SAE50}}{{k}_{SAE50}}=1+\frac{{k}_{\mathrm{ZnO}}{A}_{\mathrm{ZnO}}}{{k}_{SAE50}{A}_{SAE50}}+c{k}_{\mathrm{ZnO}}Pe\left(\frac{{A}_{\mathrm{ZnO}}}{{k}_{SAE50}{A}_{SAE50}}\right),\\ \frac{{A}_{\mathrm{ZnO}}}{{A}_{SAE50}}=\frac{{d}_{SAE50}}{{d}_{\mathrm{ZnO}}}\left(\frac{\phi }{1-\phi }\right),\\ Pe=\frac{{u}_{ZnO}{d}_{ZnO}}{{\alpha }_{\text{SAE50 }}}, { u}_{ZnO}=\frac{2{k}_{B}T}{\pi {\mu }_{SAE50}{d}_{ZnO}^{2}}, c=\mathrm{25,000}.\end{array}\right\}$$

### Similarity variables

The adopted similarity transformations are given as

$$\psi =x\sqrt{c{v}_{SAE50}}f\left(\eta \right), \eta =\sqrt{\frac{{U}_{w}\left(x\right)}{x{v}_{SAE50}}}y, u=cx{f}^{^{\prime}}\left(\eta \right), v=-\sqrt{c{v}_{SAE50}}f\left(\eta \right)$$

23 $$\theta \left(\eta \right)=\frac{T-{T}_{w}}{{T}_{w}-{ T}_{\infty }}\left(CWT\right), \theta \left(\eta \right)=\frac{T-{T}_{\infty }}{{T}_{\infty }}\left(NH\right),\Phi \left(\eta \right)=\frac{C-{C}_{w}}{{C}_{w}-{ C}_{\infty }}$$

By using similarity variables defined in Eq. (23) and taking help from Eqs. (5, 6) and (9–22), Eq. (1) satisfies identically and Eqs. (2) – (4)are reduced to nonlinear ODEs by in the following fashion. ′

24 $$\Omega_{2} \left( {e^{{ - \in_{1} \theta }} f^{\prime \prime \prime } - \in_{1} \theta^{\prime}f^{\prime\prime}} \right) - f^{\prime 2} + ff^{\prime\prime} + \Omega_{1} Q\exp \left( { - \eta \beta } \right) - \Omega_{2} \lambda e^{{ - \in_{1} \theta }} f^{\prime} - \frac{{\sigma_{ZnO - SAE50} }}{{\sigma_{SAE50} }}\Omega_{1} Mf^{\prime} - Frf^{\prime 2} = 0$$

25 $$\frac{{k_{ZnO - SAE50} }}{{k_{SAE50} }}\frac{{\Omega_{3} }}{\Pr }\left( {\left( {1 + \in_{2} \theta } \right)\theta^{\prime \prime } + \in_{2} \theta^{\prime 2} } \right) + f\theta^{\prime } + \frac{1}{\Pr }\Omega_{3} Rd\theta^{\prime \prime } - \lambda_{h} \left( {ff^{\prime } \theta^{\prime } + f^{2} \theta^{\prime \prime } } \right) - \Omega_{3} S\theta + \Omega_{4} e^{{ - \in_{1} \theta }} Ecf^{\prime \prime 2} = 0$$

26 $${\Omega }_{5} \left( {\left( {1 + \in_{3} \Phi } \right)\Phi^{\prime\prime} + \in_{3} \Phi^{{\prime}{2}} } \right) + Scf\Phi^{\prime} - Sc\lambda_{m} \left( {ff^{\prime}\Phi^{\prime} + f^{2} \Phi^{\prime\prime}} \right) - RcSc\Phi^{m} = 0$$

The converted boundary constraints are given by:

$${f}^{^{\prime}}\left(0\right)=1, f\left(0\right)=0, \theta \left(0\right)=1\left(CWT\right), {\theta }^{^{\prime}}\left(0\right)=-\delta \left(1+\theta \left(0\right)\right)\left(NH\right),\Phi \left(0\right)=0,$$

27 $${f}^{^{\prime}}\left(\infty \right)=0, \theta \left(\infty \right)=0,\Phi \left(\infty \right)=1$$

Here,

$${\Omega }_{1}=\frac{1}{\left(1-\phi \right)+\phi \left(\frac{{\rho }_{\text{ZnO }}}{{\rho }_{\text{SAE50}}}\right)}, {\Omega }_{2}=\frac{\left(1.035+0.04336\phi -0.002407T\right)}{\left(1-\phi \right)+\phi \left(\frac{{\rho }_{\text{ZnO }}}{{\rho }_{\text{SAE50}}}\right)}, {\Omega }_{3}=\frac{1}{\left(1-\phi \right)+\phi \left\{\frac{{\left(\rho {C}_{p}\right)}_{\text{ZnO }}}{{\left(\rho {C}_{p}\right)}_{\text{SAES0}}}\right\}}$$

$${\Omega }_{4}=\frac{\left(1.035+0.04336\phi -0.002407T\right)}{\left(1-\phi \right)+\phi \left\{\frac{{\left(\rho {C}_{p}\right)}_{\text{ZnO }}}{{\left(\rho {C}_{p}\right)}_{\text{SAES0}}}\right\}}, {\sigma }_{\mathrm{ZnO}-\mathrm{SAE}50}={\sigma }_{\mathrm{SAE}50}\left[1+\frac{3\left(\frac{{\sigma }_{\mathrm{ZnO}}}{{\sigma }_{\mathrm{SAE}50}}-1\right)\phi }{\left(\frac{{\sigma }_{\mathrm{ZnO}}}{{\sigma }_{\mathrm{SAE}50}}+2\right)-\left(\frac{{\sigma }_{\mathrm{ZnO}}}{{\sigma }_{\mathrm{SAE}50}}-1\right)\phi }\right]$$

and $${\Omega }_{5}={\left(1-\phi \right)}^{2.5}$$.

The non-dimensional quantities used in our study are,

$$\lambda =\frac{{v}_{SAE50}}{{k}^{*}c}, Q=\frac{\pi {j}_{0}{M}_{0}}{\delta {\rho }_{SAE50}{U}_{w}c}, \beta =\sqrt{\frac{{\pi }^{2}{v}_{SAE50}}{{p}^{2}c}}, Pr=\frac{{\mu }_{SAE50}{\left({C}_{p}\right)}_{SAE50}}{{k}_{SAE50}}, Rc=\frac{{k}_{0}{\left(C-{C}_{\infty }\right)}^{m-1}}{c}$$

$$S=\frac{{Q}_{0}}{{\rho }_{SAE50}{\left({C}_{p}\right)}_{SAE50}c}, Sc=\frac{{v}_{SAE50}}{{D}_{SAE50}}, {\lambda }_{H}=\frac{{\lambda }_{h}}{c}, {\lambda }_{M}=\frac{{\lambda }_{m}}{c}, Fr=\frac{{C}_{b}}{\sqrt{{k}^{*}}}, Rd=\frac{16{\sigma }^{*}{T}_{\infty }^{3}}{3{k}_{SAE50}{k}^{*}}$$

28 $$Ec=\frac{{U}_{w}^{2}}{{\left(Cp\right)}_{SAE50}\left({T}_{w}-{T}_{\infty }\right)}, \delta =-{h}_{s}\sqrt{\frac{{\upsilon }_{SAE50}}{c}},\mathrm{and }\,M=\frac{{\sigma }_{SAE50}{B}_{0}^{2}}{\mathrm{c}{\rho }_{SAE50}}$$

### Quantities of engineering interest

The substantial engineering coefficients are defined as follows:

$$C{f}_{x}=\frac{{\tau }_{w}}{{\rho }_{SAE50}{U}_{w}^{2}}$$

29 $$N{u}_{x}=\frac{{q}_{w}x}{{k}_{SAE50}({T}_{w}-{T}_{\infty })}, S{h}_{x}=\frac{{q}_{m}x}{{C}_{\infty }{D}_{SAE50}}$$

The terms ${\tau }_{w}$, ${q}_{w},$ and ${q}_{m}$ are given as follows:

30 $${\tau }_{w}={\left(\frac{\partial u}{\partial y}\right)}_{y=0}{\mu }_{\mathrm{ZnO}-SAE50}$$

31 $${q}_{w}={-\left.\left({k}_{SAE50}+\frac{16{\sigma }^{*}{T}_{\infty }^{3}}{3{k}^{*}}\right)\frac{\partial T}{\partial y}\right|}_{y=0}$$

32 $$q_{m} = \left( {\frac{\partial C}{{\partial y}}} \right)_{y = 0} \left( { - D_{ZnO - SAE50} } \right)$$

The non-dimensional forms of the aforementioned engineering coefficients are

33 $${Re}^{0.5}C{f}_{x}=\left(1.035+0.04336\varphi -0.002407T\right)f{^{\prime}}{^{\prime}}(0)$$

34 $${Re}^{-0.5}N{u}_{x}=-{\left(\frac{{k}_{\mathrm{ZnO}-SAE50}}{{k}_{SAE50}}+Rd\right)\theta ^{^{\prime}}}\left(0\right)$$

35 $${Re}^{-0.5}S{h}_{x}=-{\left(1-\varphi \right)}^{2.5}{\Phi }^{^{\prime}}\left(0\right)$$

Here, $Re=\frac{a{x}^{2}}{{v}_{SAE50}}$ is the local Reynolds number.

## Solution methodology

The governing system of PDEs has been altered to the system of coupled ODEs with the introduction of apposite similarity variables. The resulting system of coupled ODEs, as given in Eqs. (24, 25, 26)  along-with the most appropriate BCs given in Eq. (27), has been treated numerically by using MATLAB bvp-4c solver.

### Introduction to Bvp-4c

To execute the BVP of the form $\eta^{\prime} = f\left( {\xi ,\eta ,c} \right)$, with two point BC $\psi \left( {\eta \left( a \right),\eta \left( b \right),c} \right) = 0$ where $a\le \xi \le b$, bvp-4c applies a collection method. The following two key steps are involved in the methodology^51^.

### Step $1:$ Approximate solution

Adequate the BCs $\psi \left(g\left(a\right),g\left(b\right)\right)=0,$ a polynomial $g(x)$ is well-defined over each interval $\left[{\xi }_{n},{ \xi }_{n+1}\right]$ of each mesh $a={\xi }_{0}<{\xi }_{1}<\dots <{\xi }_{n}=b$. Furthermore, at both ends and mid points of each subinterval $g(\xi )$ satisfies the collocates i.e.,

36 $${g}^{^{\prime}}\left({\xi }_{n}\right)=f({\xi }_{n},g\left({\xi }_{n}\right))$$

37 $${g}^{^{\prime}}\left(\frac{{\xi }_{n}+{\xi }_{n+1}}{2}\right)=f\left(\frac{{\xi }_{n}+{\xi }_{n+1}}{2}, g\left(\frac{{\xi }_{n}+{\xi }_{n+1}}{2}\right)\right)$$

and

38 $${g}^{^{\prime}}\left({\xi }_{n+1}\right)=f\left({\xi }_{n+1},g\left({\xi }_{n+1}\right)\right)$$

By Simpson’s method, these are iteratively worked out. To an isolated solution $\eta (\xi )$, $g(\xi )$ may be a $4th$ order estimate with a small premise.

39 $$\Vert \eta \left(\xi \right)-g(\xi )\Vert \le {Ch}^{4}$$

where, $C$ and $h$ represent the constant and the step size ${h}_{n}={\xi }_{n+1}-{\xi }_{n}$, respectively. At any $\xi$ in $[a,b]$, $g(\xi )$ can be found inexpensively with ‘‘bvpval’’ function, since it is executed on a mesh with bvp-4c.

### Step $2:$ Residual function ${\varvec{R}}({\varvec{\xi}})$

Define residual in ODEs as $R\left( \xi \right) = g^{\prime}\left( \xi \right) - f\left[ {\xi ,g\left( \xi \right)} \right]$ in correspondence with the BC $\psi \left(g\left(a\right),g\left(b\right)\right)=0$, to minimize the error. $g(\xi )$ is an excellent solution, if the residuals are small uniformly.

### Numerical solution with MATLAB Bvp4c solve

In system of Eqs. (24) − (27), we introduce the following substitutions,

$${y}_{1}=f, { y}_{2}={f}^{^{\prime}} , {y}_{3}={f}^{{^{\prime}}{^{\prime}}{^{\prime}}} , {y}_{3}^{^{\prime}}={f}^{{^{\prime}}{^{\prime}}{^{\prime}}} , {y}_{4}=\theta , {y}_{5}={\theta }^{^{\prime}}, { y}_{5}^{^{\prime}}={\theta }^{{^{\prime}}{^{\prime}}} ,$$

$${y}_{6}=\Phi , {y}_{7}={\Phi }^{^{\prime}} , { y}_{7}^{^{\prime}}={\Phi }^{{^{\prime}}{^{\prime}}}.$$

The system of higher order equations have been reduced to first order along with boundary conditions as:

40 $${y}_{3}^{^{\prime}}=\frac{1}{{\Omega }_{2}{e}^{-{\epsilon }_{1}{y}_{4}}}({\Omega }_{2}{\epsilon }_{1}{y}_{5}{y}_{3}+{y}_{2}^{2}-{y}_{1}{y}_{3}-{\Omega }_{1}Q\mathrm{exp}\left(-\eta \beta \right)-{\Omega }_{2}\lambda {e}^{-\epsilon {y}_{4}}{y}_{2}+Fr{y}_{2}^{2} +\left(\frac{{\sigma }_{\mathrm{ZnO}-SAE50}}{{\sigma }_{SAE50}}\right){\Omega }_{1}M{y}_{2}$$

$${y{^{\prime}}}_{5}=\frac{1}{{\Omega }_{3}\left(\left(\frac{{k}_{\mathrm{ZnO}-SAE50}}{{k}_{SAE50}}\right)\left(1+{\epsilon }_{2}{y}_{4}\right)+Rd-{\lambda }_{h}Pr{y}_{1}^{2}\right)}(-{\Omega }_{3}{\epsilon }_{2}\left(\frac{{k}_{\mathrm{ZnO}-SAE50}}{{k}_{SAE50}}\right){y}_{5}^{2}-{y}_{1}{y}_{5}Pr$$

41 $$+{\lambda }_{h}Pr{y}_{1}{y}_{2}{y}_{5}+{\Omega }_{3}PrS{y}_{4}-{\Omega }_{4}{e}^{-{\epsilon }_{1}{y}_{4}}PrEc{y}_{3}^{2})$$

42 $${y{^{\prime}}}_{7}=\frac{1}{{\Omega }_{5}\left(1+{\epsilon }_{2}{y}_{6}\right)-{\lambda }_{m}Sc{y}_{1}^{2}}\left(-{\Omega }_{5}{\epsilon }_{3}{y}_{7}^{2}-Sc{y}_{1}{y}_{7}+{\lambda }_{m}Sc{y}_{1}{y}_{2}{y}_{7}+ScRc{y}_{6}^{n}\right)$$

43 $$\left\{\begin{array}{c}{y}_{2}\left(0\right)=1, {y}_{1}\left(0\right)=0, {y}_{4}\left(0\right)=1 \left(CWT\right),\\ \\ {y}_{5}\left(0\right)=-\delta \left(1+{y}_{4}\left(0\right)\right) \left(NH\right), {y}_{6}\left(0\right)=0,\\ \\ {y}_{2}\left(\infty \right)=0, {y}_{4}\left(\infty \right)=0, {y}_{6}\left(\infty \right)=0. \end{array}\right.$$

On the residuals, the absolute and relative errors of tolerance have been set by

44 $$options=bvpset({^{\prime}}{^{\prime}}AbsTo{l}^{{^{\prime}}{^{\prime}}}, le-8{,}^{^{\prime}}Re lTo{l}^{^{\prime}},le-5)$$

The resulting IVPs have been solved by $^{\prime\prime}bvpinit^{\prime\prime}$

45 $$solinit=bvpinit\left(linespace\left(0, 5, 50\right),\left[\mathrm{0,5}\right]\right)$$

46 $$solinit=bvpinit\left(linespace\left(0, 8, 50\right),\left[\mathrm{0,8}\right]\right)$$

and

47 $$solinit=bvpinit\left(linespace\left(0, 7, 50\right),\left[\mathrm{0,7}\right]\right)$$

The BVP alongwith the BCs has been executed by following code:

48 $$sol=bvp4c(file\_ode, file\_bc,solinit,options)$$

The following syntax has been used to calculate the residual:

49 $$res=file\_bc(ya,yb)$$

## Results and discussion

The motive behind this section is to explain the graphical and numerical outcomes of various important parameters of interest on concerned profiles of nanolubricant ${\mathrm{ZnO}-SAE50}$. The governing system of PDEs alongwith B.Cs is reduced to ODEs by applying apposite similarity transformations. The altered system of ODEs alongwith B.Cs is treated numerically by using MATLAB built-in bvp4c package. The results have been plotted to assess the behaviors of concerned profiles corresponding to the variation of numerous parameters of interest. The values of the parameters have been taken as $0.1\le \lambda \le 1, 0.1\le Q\le 0.7, 0.2\le \beta \le 1.5, 0.1\le M\le 1.5, 0.0025\le \phi \le 0.0025, 0.1\le Fr\le 1.3, 0.1\le {\in }_{1}\le 2, 0.1\le Rd\le 4, 0.1\le Ec\le 1.5, -0.3\le S\le 1, -0.7\le {\in }_{2}\le -0.1, 0.1\le {\lambda }_{h}2.0, 0.1\le {\in }_{3}\le 0.8, 0.1\le Rc\le 1.5, 1\le m\le 3, 0.5\le Sc\le 2.0, 0.1\le {\lambda }_{m}1.0.$ The thermo-physical physical properties of $SAE50$ and ${\mathrm{ZnO}}$ are presented in Table 1.

**Table 1:** The features of $SAE50$ (base fluid) and ${\mathrm{ZnO}}$ (nanoparticles)^49^.

S. no.		${\mathrm{ZnO}}$	$SAE50$
$1$	$\rho \left( {{\text{Kg}}/{\text{m}}^{3} } \right)$	$5.606$	$0.906$
$2$.	$C_{p} \left( {{\text{J}}/{\text{KgK}}} \right)$.	$544$	$1900$
$3$	$k\left( {{\text{W}}/{\text{mK}}} \right)$	$19$	0.15
$4$	$\mu f\left( {{\text{Ns}} \cdot {\text{m}}^{2} } \right)$	$-$	0.192543
5	$df/ds\left( {{\text{nm}}} \right)$	60	40
6	$\sigma$	0.01	–

### Velocity Profile

**Figure 2:**
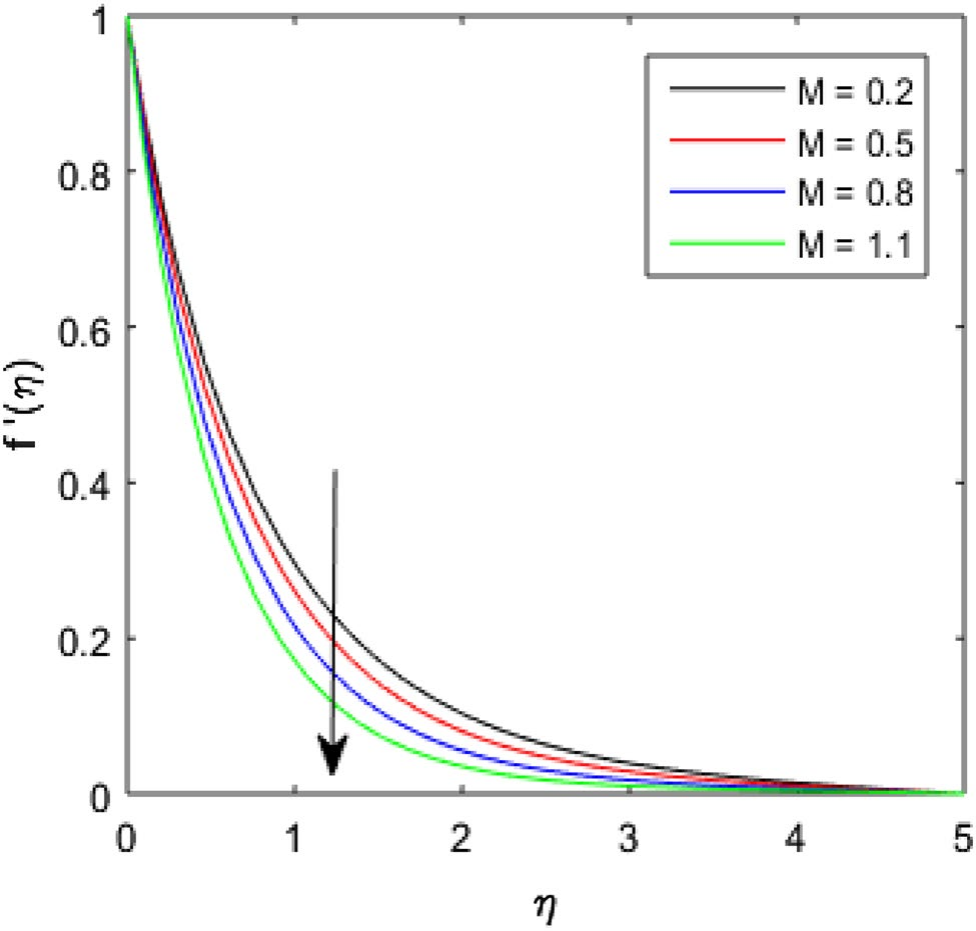
$f^{\prime}\left( \eta \right)$ verses varied values of $M$.

**Figure 3:**
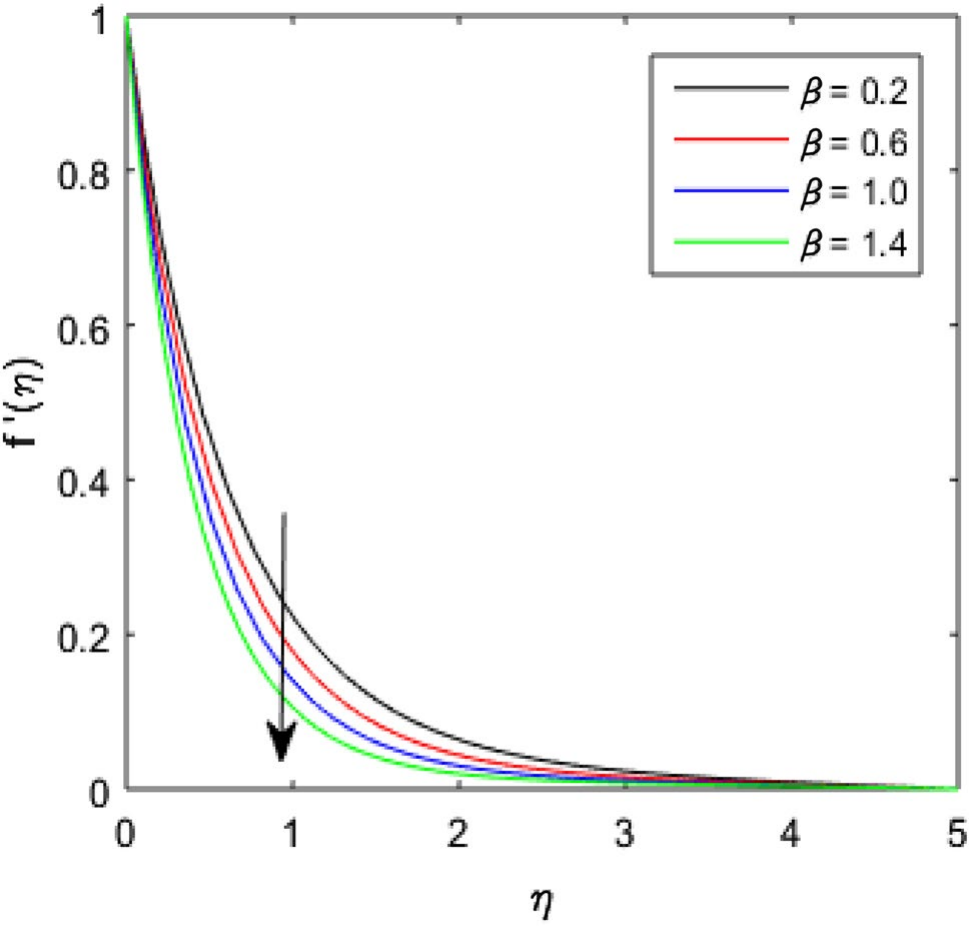
$f^{\prime}\left( \eta \right)$ verses varied values of $\beta$.

**Figure 4:**
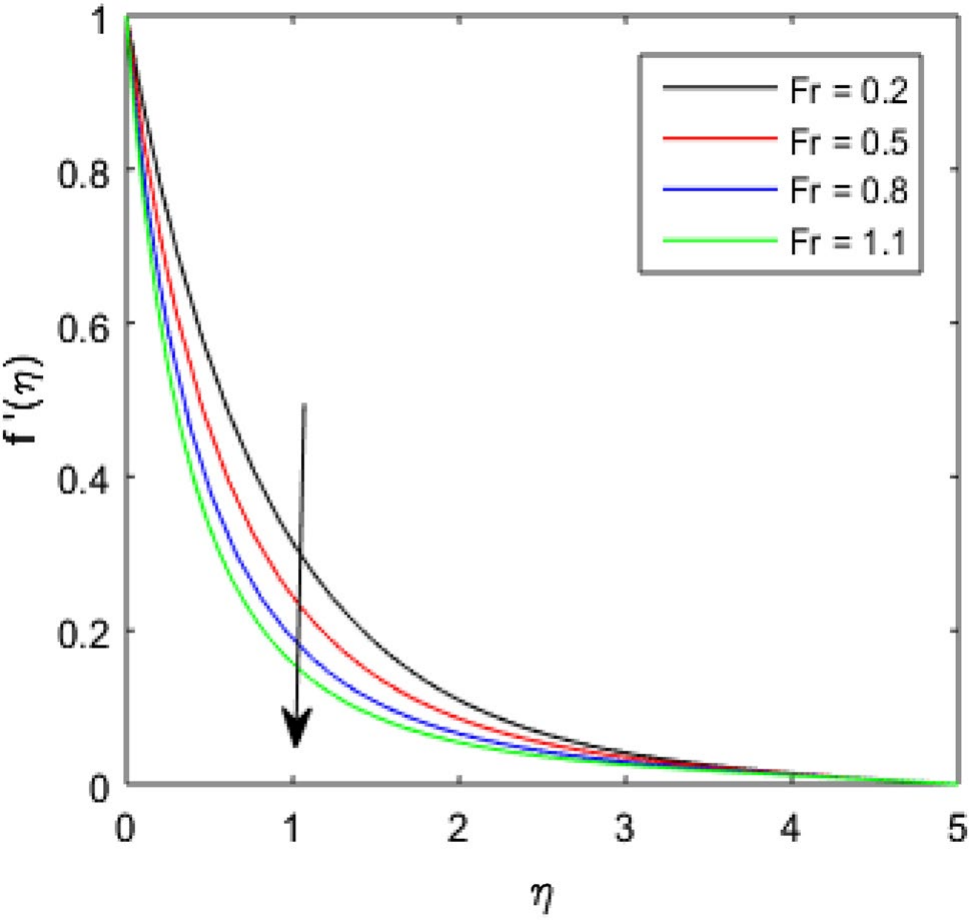
$f^{\prime}\left( \eta \right)$ verses varied values of $Fr$.

**Figure 5:**
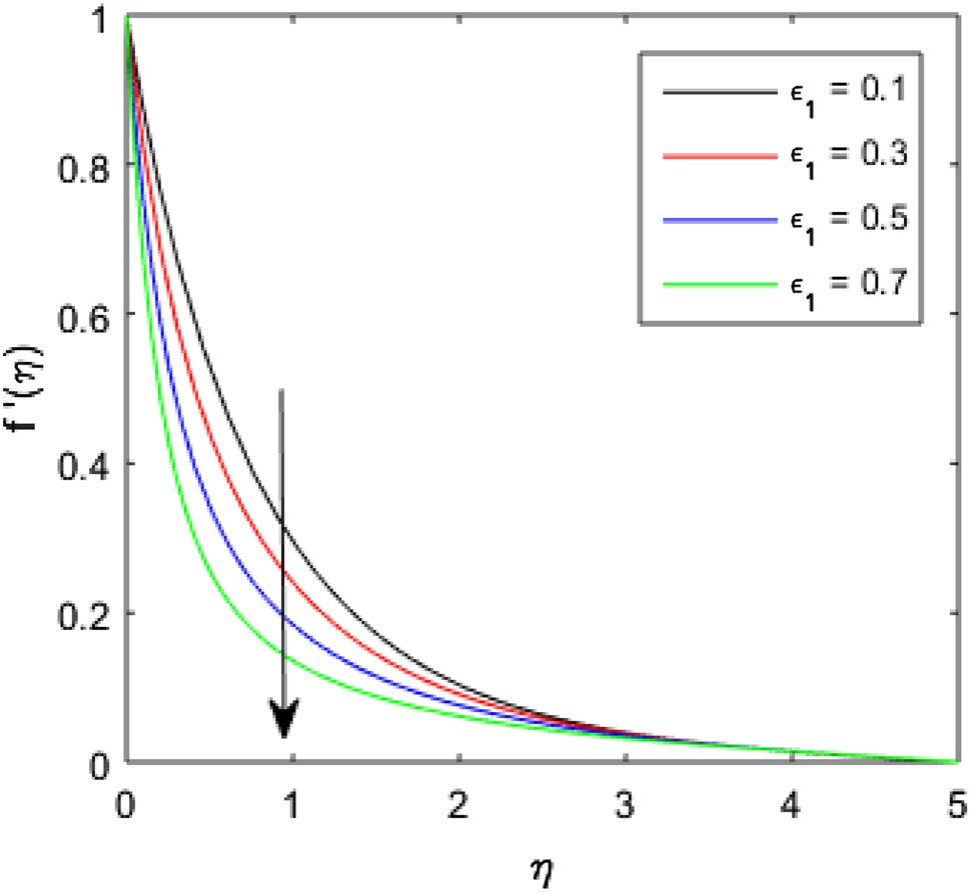
$f^{\prime}\left( \eta \right)$ verses varied values of $\in_{1}$.

**Figure 6:**
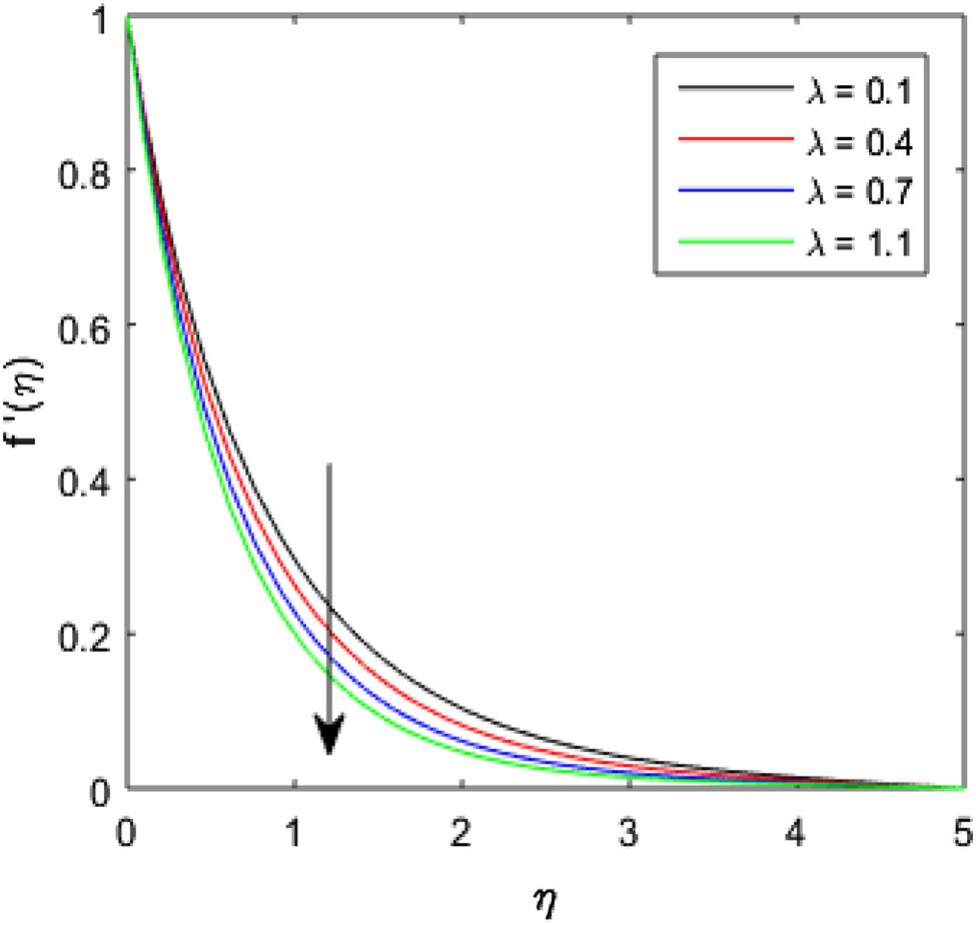
$f^{\prime}\left( \eta \right)$ verses varied values of $\lambda$.

**Figure 7:**
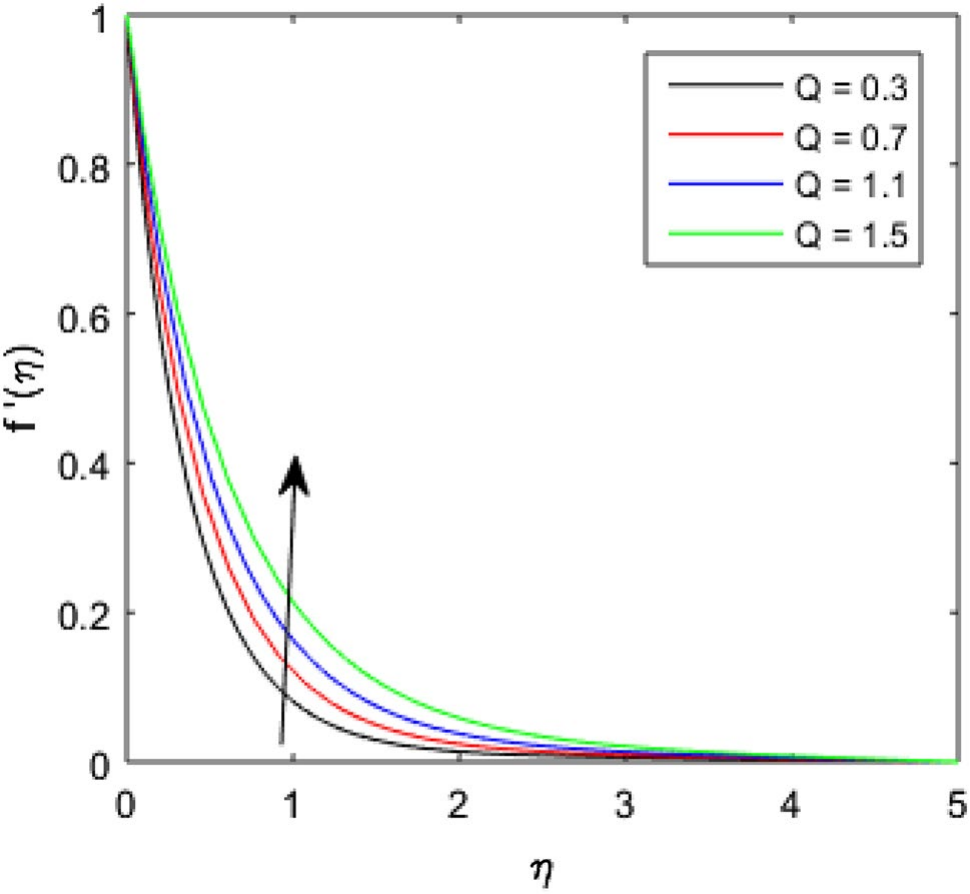
$f^{\prime}\left( \eta \right)$ verses varied values of $Q$.

**Figure 8:**
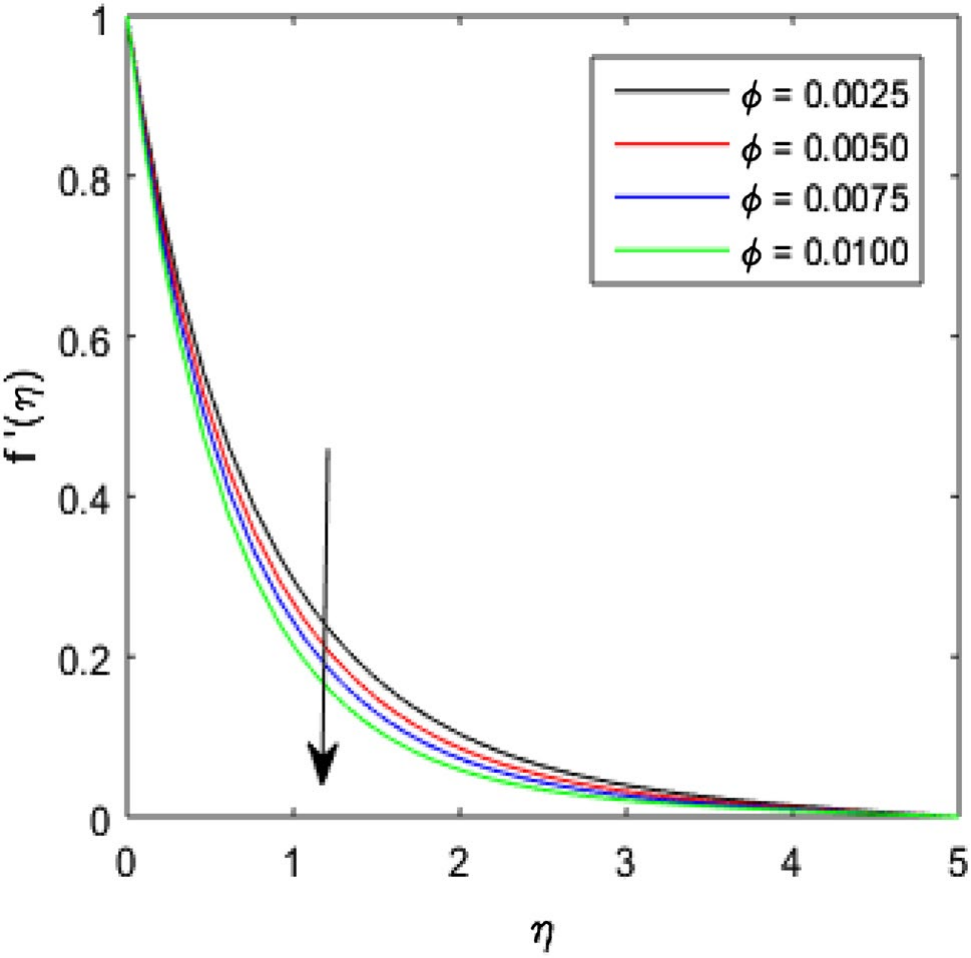
$f^{\prime}\left( \eta \right)$ verses varied values of $\phi$.

To describe the effects of magnetic parameter $M$, inertial coefficient $Fr$, viscosity parameter ${\epsilon }_{1}$, porosity parameter $\lambda$, modified Hartmann number $Q$, width parameter $\beta$ and solid volume fraction parameter $\phi$ on velocity $f^{\prime}\left( \eta \right)$ of the nanolubricant by keeping other parameters fixed, Figs. 2, 3, 4, 5, 6, 7, 8 are sketched and their preliminary performances are assessed. The impact of magnetic parameter $M$ on velocity $f^{\prime}\left( \eta \right)$ of the nanolubricant is revealed in Fig. 2. The retardation in the velocity of the nanolubricant is caused due to growing magnetic field’s strength. This retardation occurs just because of the Lorentz force produced during strengthening the magnetic field. Figure 3 explains the behavior of velocity profile $f^{\prime}\left( \eta \right)$ against varied values of $\beta$. The velocity profile decays by augmenting $\beta$. The impact of inertial parameter $Fr$ on the velocity profile $f^{\prime}\left( \eta \right)$ is demonstrated in Fig. 4. The rise in $Fr$ reduces the velocity $f^{\prime}\left( \eta \right)$ of the nanolubricant. A fall in the velocity profile happens because the higher $Fr$ increases thickness of the boundary. Figure 5 discloses the impact of $\in_{1}$ on velocity profile $f^{\prime}\left( \eta \right)$. The velocity of the nanolubricant gets decreased as viscosity parameter $\in_{1}$ upsurges. This is mostly explained by the fact that increasing viscosity widens the boundary layer, which lessens the velocity of the nanolubricant. The upshot of velocity profile $f^{\prime}\left( \eta \right)$ versus rising values of porosity parameter $\lambda$ is presented in Fig. 6. A rise in the values of $\lambda$ causes a decline in the nanolubricant flow's velocity distribution. The frictional drag force created by resistance caused by porous space opposes the liquid flow's velocity, causing the velocity of the nanolubricant to decrease. The effect of $Q$ on the velocity profile $f^{\prime}\left( \eta \right)$ is exposed in Fig. 7. The velocity of the nanolubricant gets accelerated by increasing the values of $Q$. This enhanced response arises because along the plate, the flow is reinforced by the Lorentz force parallel to surface of the Riga plate. Figure 8 explains the response of velocity profile $f^{\prime}\left( \eta \right)$ of the nanolubricant against varied values of $\phi$. The enhancing values of $\phi$ minimize the velocity of nanolubricant. Physically, the boundary layer thickness gets improved with the addition of more nanoparticles, which results a decline in the velocity of the nanolubricant.

### Temperature profile

**Figure 9:**
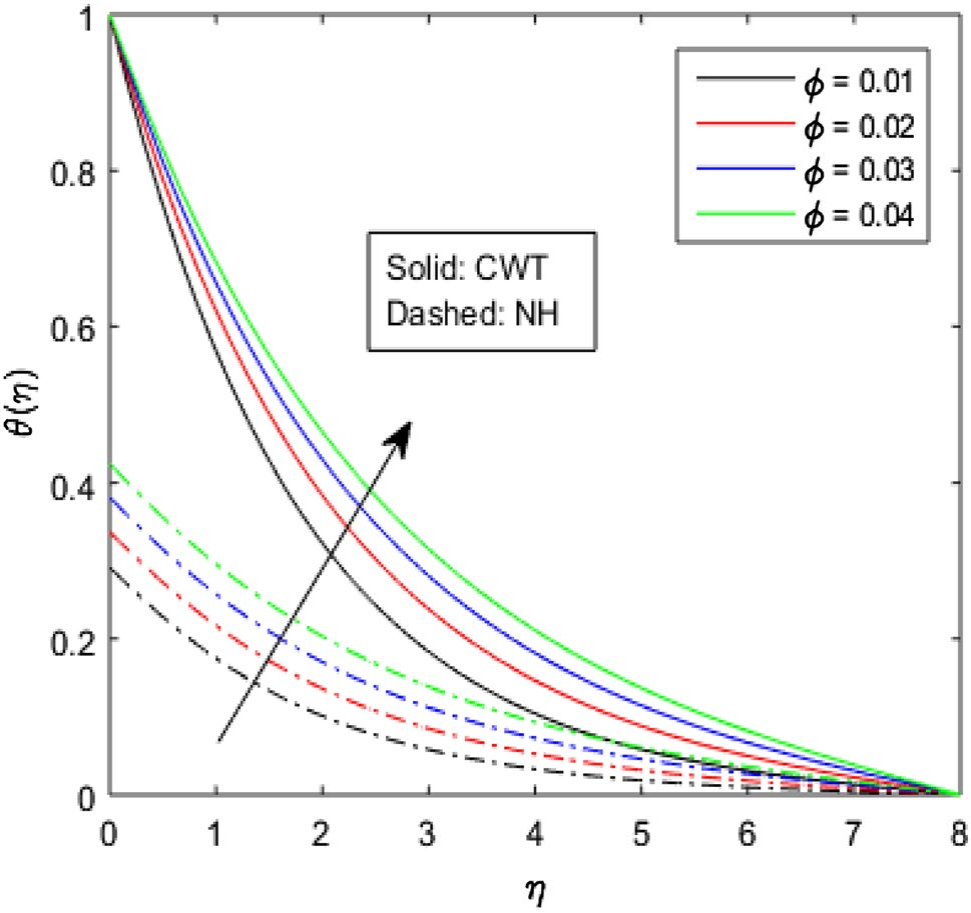
$\theta (\eta )$ verses varied values of $\phi$.

**Figure 10:**
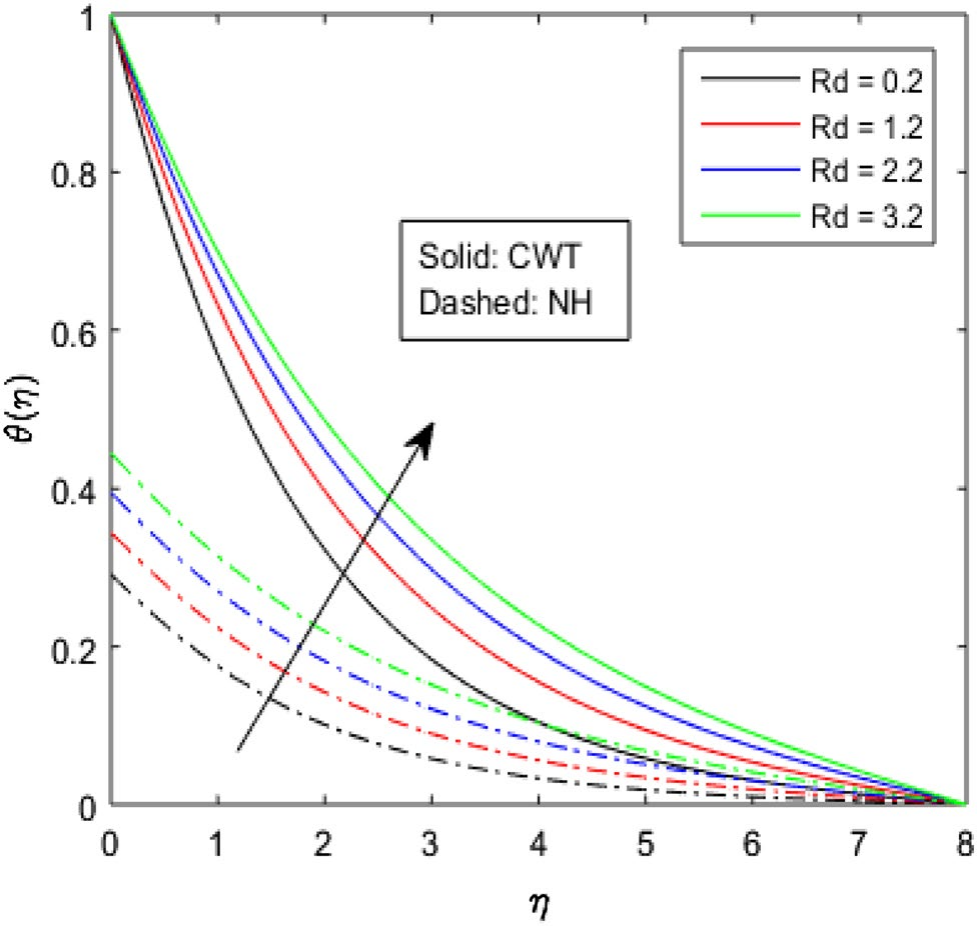
$\theta \left(\eta \right)$ verses varied values of $Rd$.

**Figure 11:**
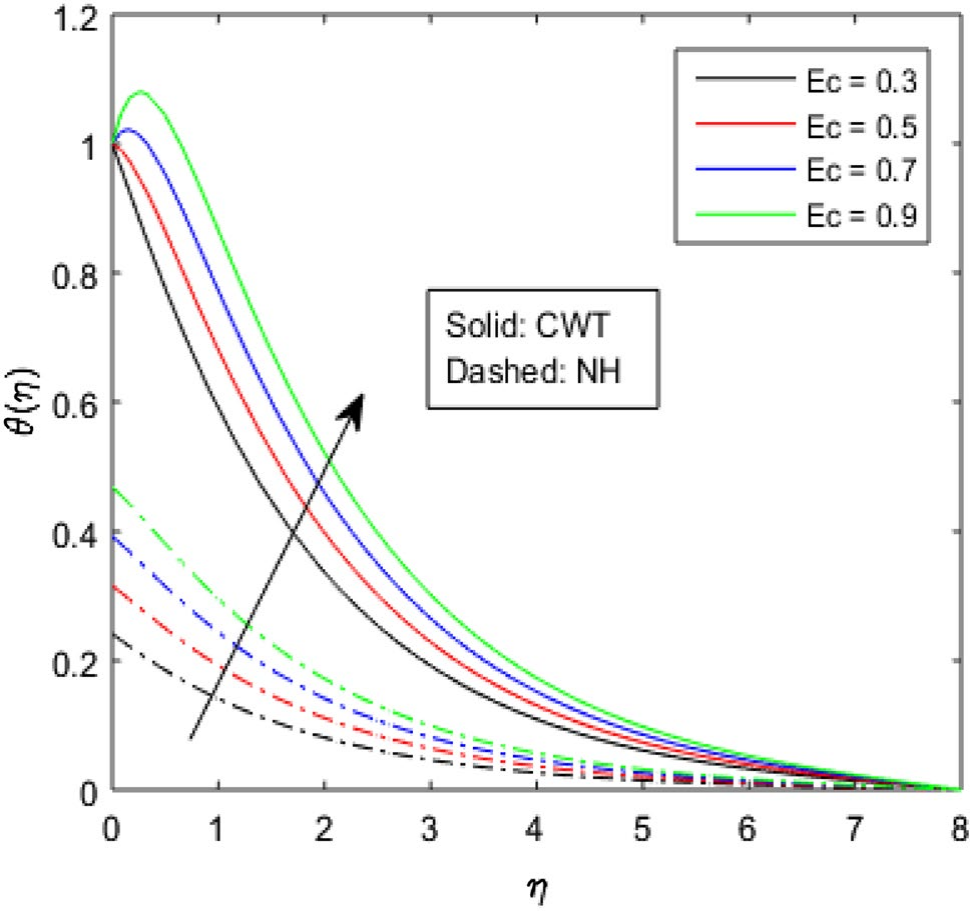
$\theta (\eta )$ verses varied values of $Ec$.

**Figure 12:**
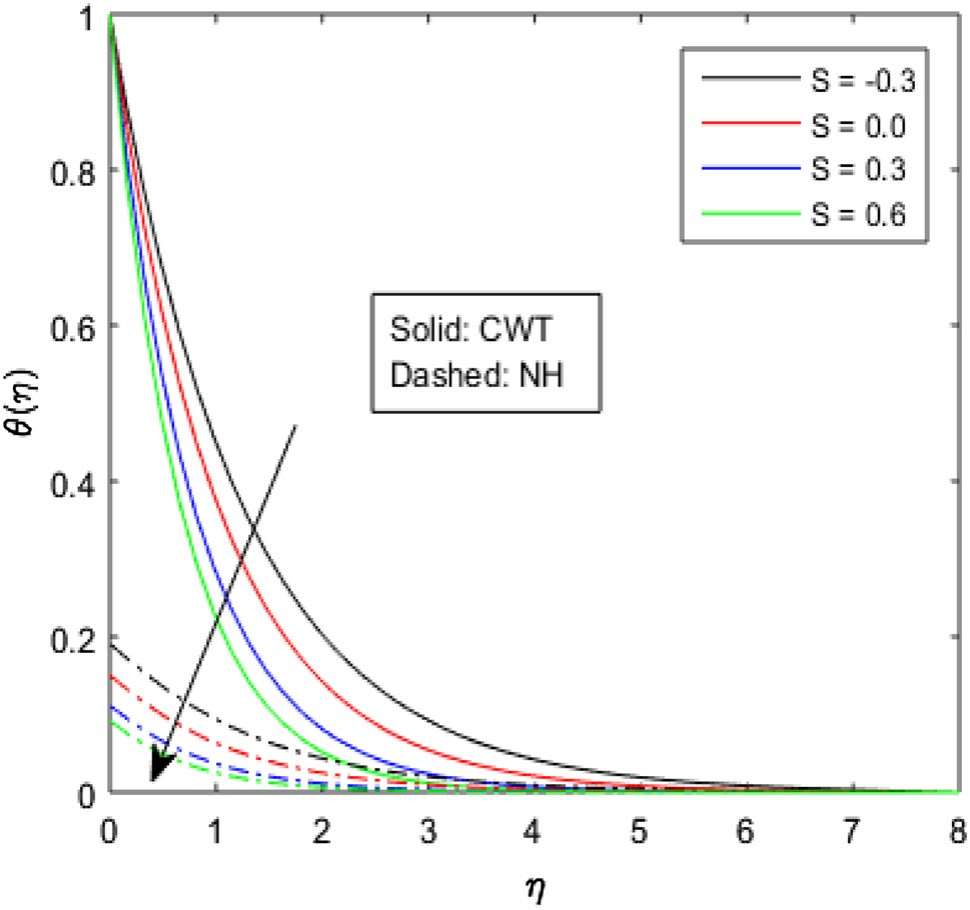
$\theta (\eta )$ verses varied values of $S$.

**Figure 13:**
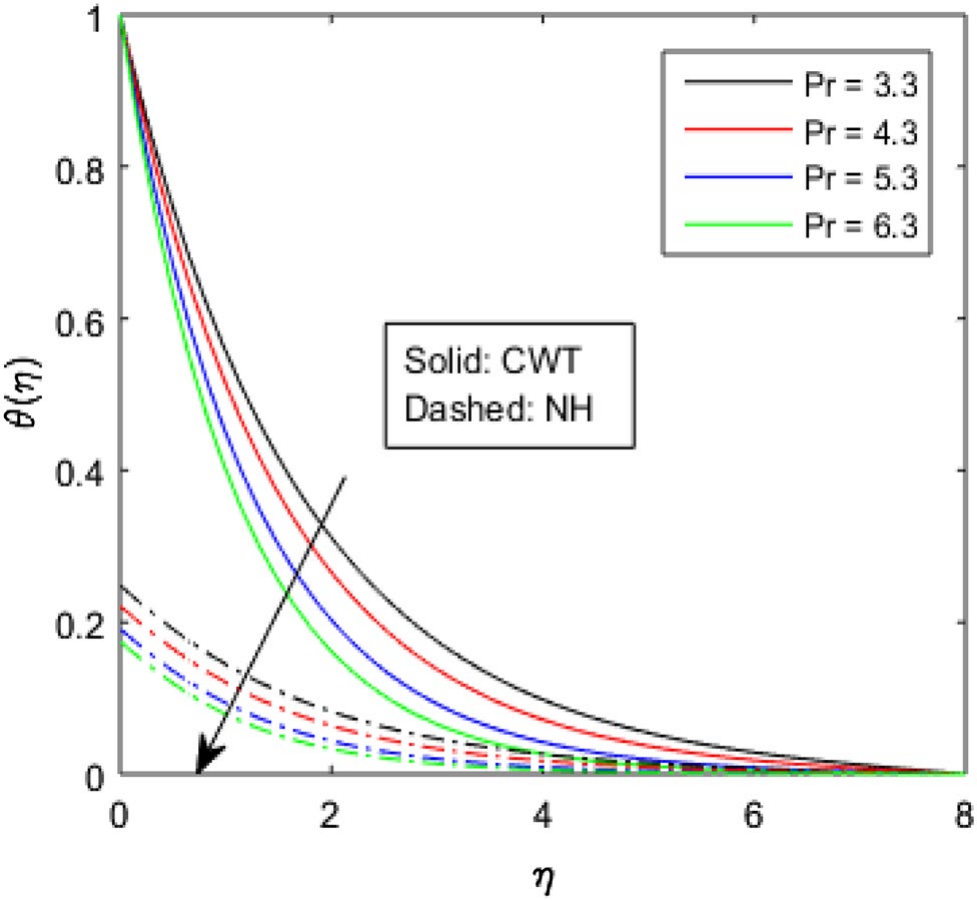
$\theta (\eta )$ verses varied values of $Pr$.

**Figure 14:**
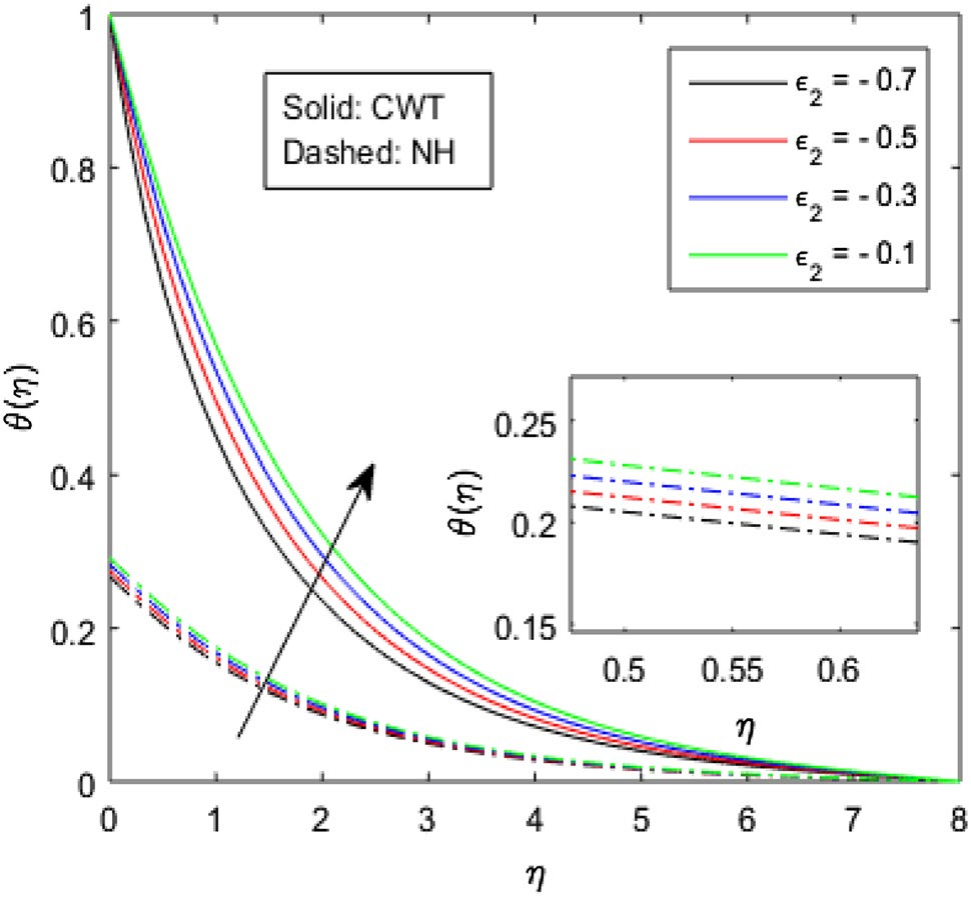
$\theta (\eta )$ verses varied values of ${\epsilon }_{2}$.

**Figure 15:**
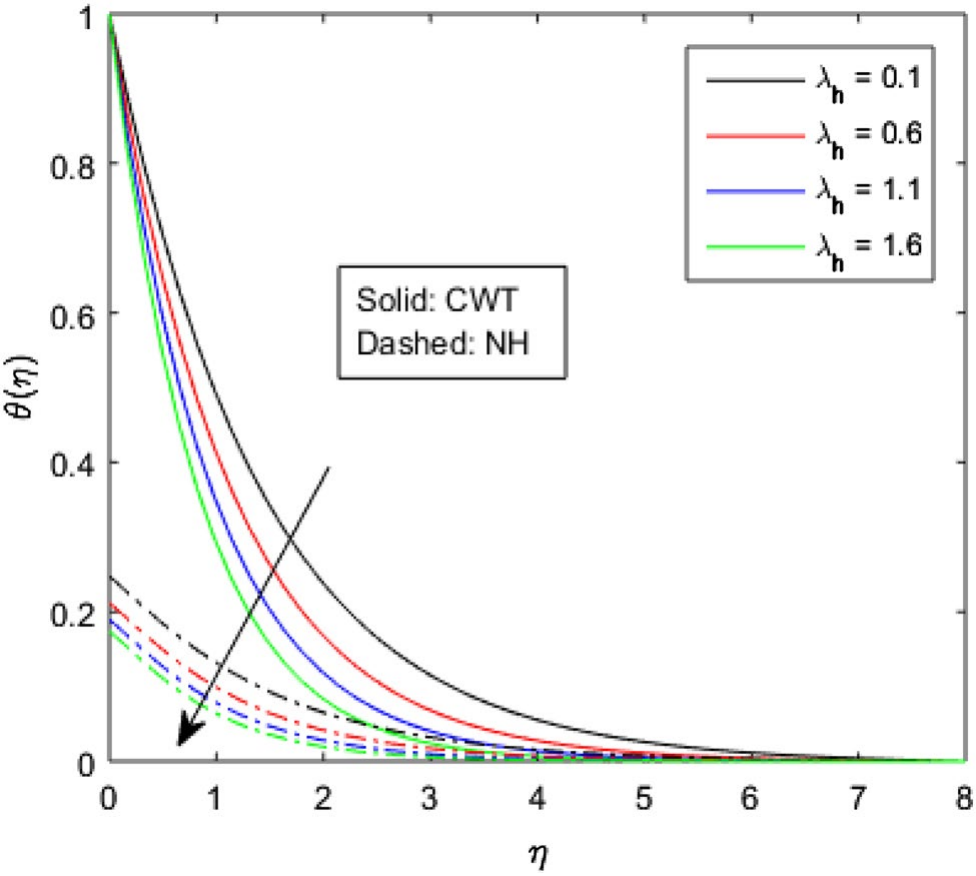
$\theta (\eta )$ verses varied values of ${\lambda }_{h}$.

Figures 9, 10, 11, 12, 13, 14 and 15 have been plotted to assess the outcomes of temperature profile $\theta (\eta )$ against dissimilar values of solid volume fraction $\phi$, radiation parameter $Rd$, Eckert number $Ec$, heat source/sink parameter $S$, Prandtl number $Pr$, thermal conductivity parameter ${\epsilon }_{2}$ and thermal relaxation parameter ${\lambda }_{h}$. The influence of $\phi$ on the temperature of the nanolubricant for constant wall temperature (CWT) and Newtonian heating (NH) situations has been visualized in Fig. 9. The nanolubricant’s temperature significantly goes up for both the situations (Newtonian heating and constant wall temperature) with an increase in the solid volume fraction $\phi$. The heat absorption capacity and viscosity of the nanolubricant get enhanced by adding more nanoparticles and heat is rapidly conveyed between the particles, as a result temperature of the nanolubricant enhances. Figure 10 elaborates the impact of radiation parameter $Rd$ on the temperature profile $\theta (\eta )$ for NH and CWT conditions. It has been found that the temperature of the nanolubricant increases in both circumstances (NH and CWT) for higher values of $Rd$. Physically, the nanolubricant absorbs radiations when radiation phenomenon is employed, and thus heat energy of the nanolubricant enhances. Therefore, the temperature profile $\theta \left(\eta \right)$ experiences a tremendous increment for higher values of $Rd$. The effect of Eckert number $Ec$ on the temperature profile $\theta (\eta )$ for NH and CWT states is demonstrated in Fig. 11. The rise in temperature of the nanolubricant is noticed by rising the values of $Ec$. The kinetic energy of the nanolubricant overpowers the amount of enthalpy produced when we enlarge $Ec$. As a result, temperature of the nanolubricant boosts for both the cases (NH and CWT) significantly in entire flow domain. Figure 12 represents the nature of temperature profile $\theta (\eta )$ for NH and CWT cases by varying heat sink/source parameter $S$. Here, $S=0$, $S<0$ and $S>0$ demonstrate the absence of heat source-sink and the existence of heat sink and source respectively. By emerging the values of $S$, the temperature of the nanolubricant increases. By virtue of the exchanger that the heat sink serves as, heat generated by the surface will be transported into the nanolubricant. As a result, in heat sink case, the thermal distribution is low, and in heat source case, the surface produces the temperature. The existence of a heat source represents better thermal performance for both the cases (Newtonian heating and constant wall temperature) than a heat sink. The change in the temperature profile $\theta (\eta )$ with enhancing $Pr$ for NH and CWT conditions has been sketched in Fig. 13. The temperature of the nanolubricant drops for greater $Pr$. This happens because for higher values of $Pr$ thermal diffusivity of the nanolubricant gets decreased, which causes the temperature profile $\theta \left(\eta \right)$ to drop. Figure 14 visualizes the impact of ${\epsilon }_{2}$ on temperature profile $\theta (\eta )$ for both the cases (NH and CWT). The heating phenomenon is effectively supported by increasing the values of thermal conductivity parameter. The use of materials with variable thermal property is found to be an aid in accelerating heat transmission. The response of the temperature profile $\theta (\eta )$ corresponding to thermal relaxation parameter ${\lambda }_{h}$ for NH and CWT situations has been demonstrated in Fig. 15. The sketch shows that temperature of the nanolubricant consequently falls for both the cases (NH and CWT) with the enhancing values of thermal relaxation parameter ${\lambda }_{h}$. Physically, a drop in the related boundary layer thickness is revealed by growing ${\lambda }_{h}$, by which the temperature of the nanolubricant drops.

### Concentration profile

**Figure 16:**
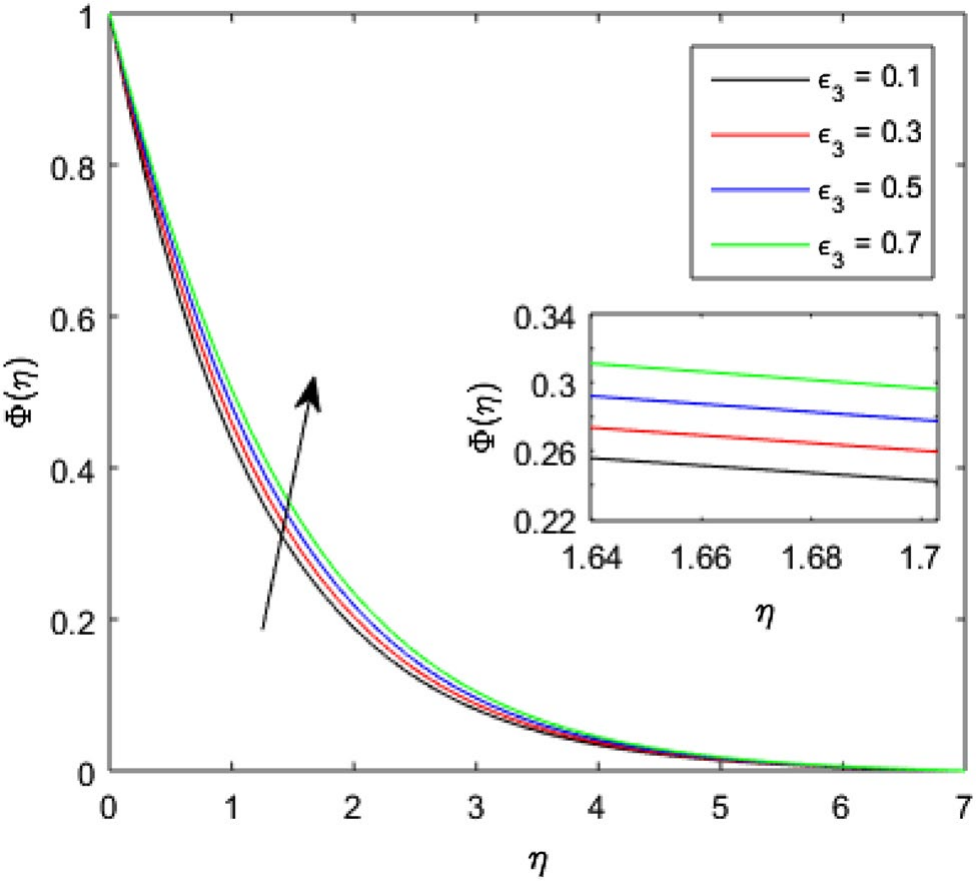
$\Phi (\eta )$ verses varied values of ${\epsilon }_{3}$.

**Figure 17:**
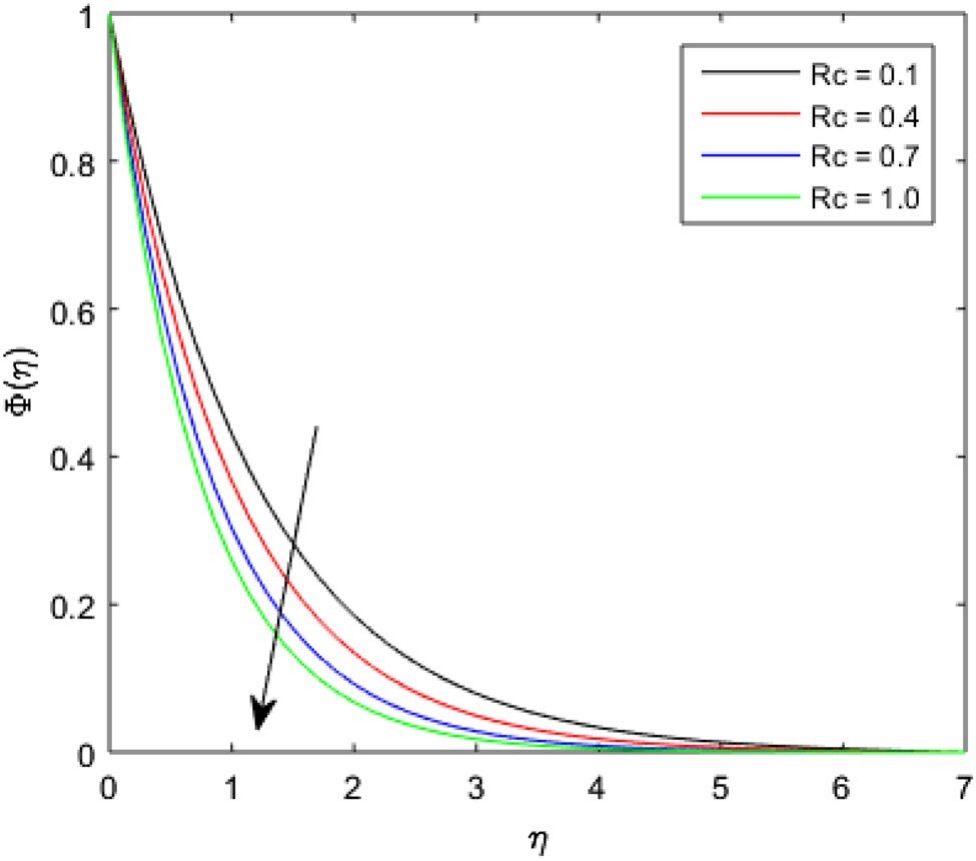
$\Phi (\eta )$ verses varied values of $Rc$.

**Figure 18:**
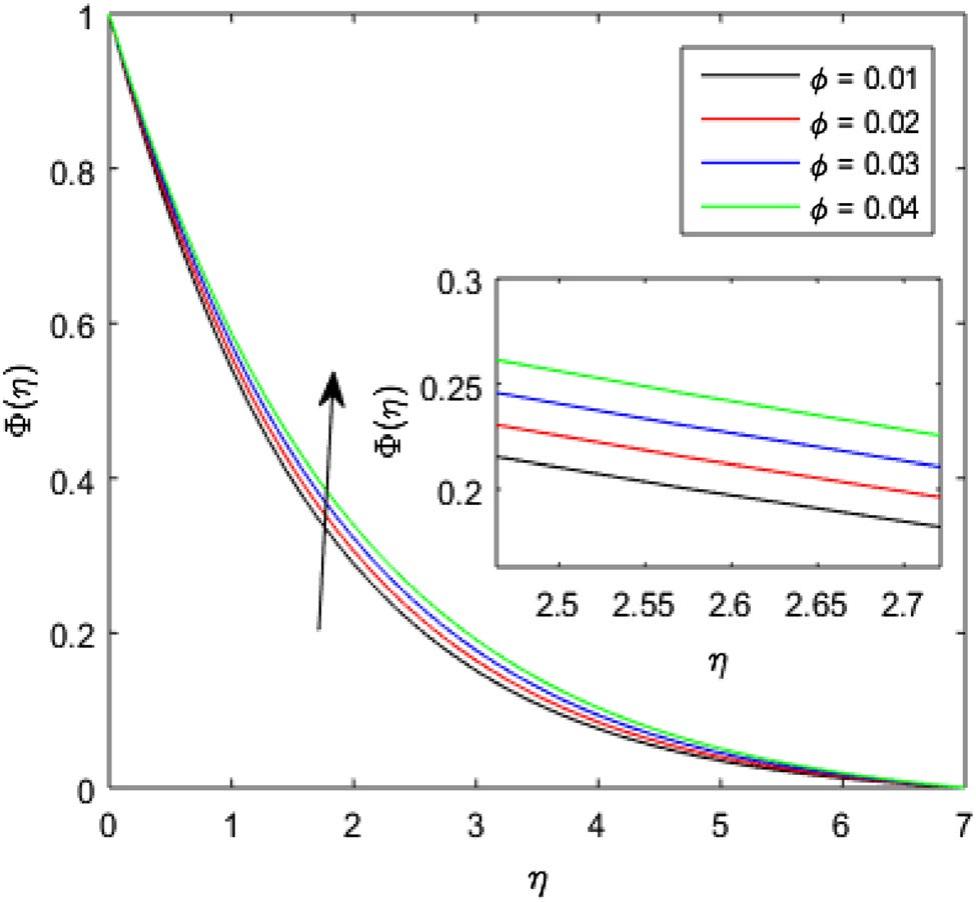
$\Phi \left(\eta \right)$ verses varied values of $\phi$.

**Figure 19:**
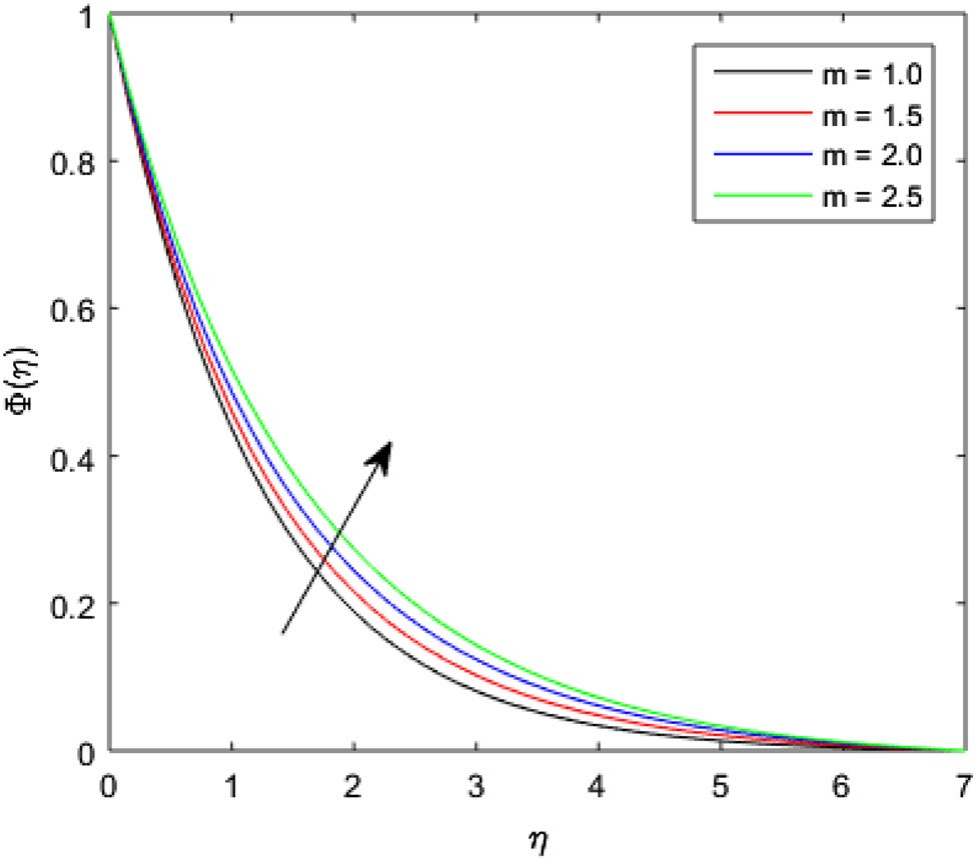
$\Phi (\eta )$ verses varied values of $m$.

**Figure 20:**
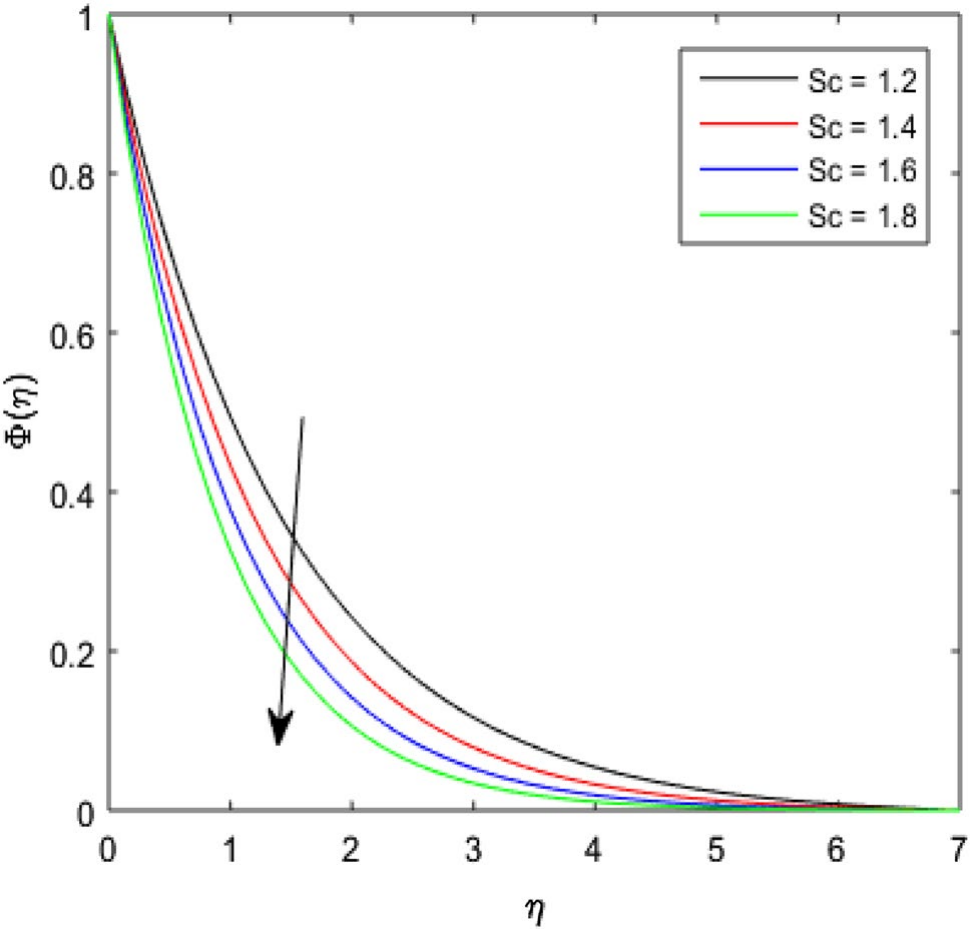
$\Phi (\eta )$ verses varied values of $Sc$.

**Figure 21:**
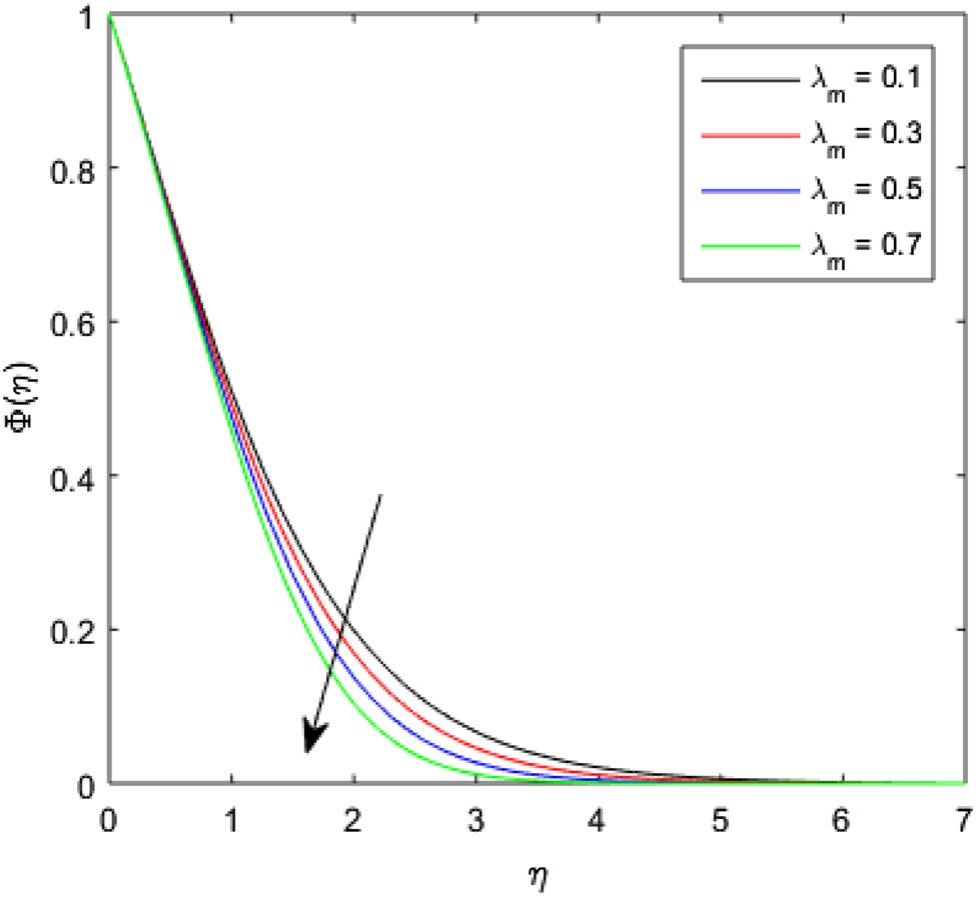
$\Phi (\eta )$ verses varied values of ${\lambda }_{m}$.

The nature of concentration profile $\Phi (\eta )$ corresponding to varied values of mass diffusivity parameter ${\epsilon }_{3}$, chemical reaction parameter $Rc$, solid volume fraction $\phi$, order of the chemical reaction $m$, Schamidt number $Sc$ and solutal relaxation parameter ${\lambda }_{m}$ has been exposed in Figs. 16, 17, 18, 19, 20 and 21. Figure 16 discloses the feature of mass diffusivity parameter ${\epsilon }_{3}$ on concentration distribution. The growth in the concentration field is reported for higher variation of mass diffusivity parameter ${\epsilon }_{3}$. Physically, the mass diffusivity gets enlarged corresponding to higher mass diffusivity parameter, which results in more transfer of mass from the Riga surface to the nanolubricant. Thus, the enhancement in solutal transport is obvious to occur. The viscosity of the nanolubricant gets lowered with an augmentation in the chemical reaction parameter $Rc$. This phenomenon takes place because of accidental entropy production in the nanolubricant. Hence, the concentration profile $\Phi \left(\eta \right)$ diminishes by rising $Rc$ as have been demonstrated in Fig. 17. In Fig. 18, the behavior of concentration profile $\Phi \left(\eta \right)$ for increasing solid volume fraction $\phi$ is executed. The concentration distribution upsurges by adding more nanoparticles to the base lubricant. Physically, an improvement in the boundary layer causes the concentration distribution to enlarge due to the accumulation of more nanoparticles in the base lubricant. Thus, the significant enhancement in the mass transfer occurs by increasing solid volume fraction. The altered profile of concentration distribution in correspondence to developing $m$ is given in Fig. 19. It is obvious to note that the concentration profile $\Phi \left(\eta \right)$ gets stronger as order of the chemical reaction goes up. The impact of $Sc$ on concentration profile $\Phi \left(\eta \right)$ is highlighted in Fig. 20. It is found that an escalation in $Sc$ diminishes the concentration distribution. This diminishment in the concentration distribution is resulted because by the escalation in $Sc$, momentum diffusivity leads the mass diffusivity. Figure 21 exposes the variation of concentration profile $\Phi \left(\eta \right)$ with the growing values of solutal relaxation parameter ${\lambda }_{m}$. The plot shows that enhancing values of solutal relaxation parameter ${\lambda }_{m}$ weaken the concentration distribution of the nanolubricant. Physically, a drop in the associated concentration boundary layer is discovered in correspondence to accumulative solutal relaxation parameter ${\lambda }_{m}$, by which the concentration distribution gets weakened.

### Tables discussion

The comparison of present model with the works published earlier, has been given in Table $1$. It is pertinent to note that the present model finds an excellent agreement with the literature. Table 2 represents the comparison for computational values of f"(0) for limited case with the literature. Table 3 represents the impacts of various parameters on Skin friction. It is observed that the value of Skin friction is less in the case of nanolubricant ${\mathrm{ZnO}-SAE50}$ as compared to $SAE50$ oil. The impacts of different parameters on Nusselt number are given in Table 4. It is detected that in common wall temperature condition (CWT), greater thermal enhancement is achieved as compared to Newtonian heating (NH) case. Table 5 shows the effects of some parameters on Sherwood number. It is noted that nanolubricant ${\mathrm{ZnO}-SAE50}$ offers better mass transfer rate in comparison to $SAE50$ oil.

**Table 2:** Model comparison for computational values of $f^{\prime\prime}\left( 0 \right)$ for limited case.

Parameter	Akbar et al. ^52^	Chu et al. ^14^	Present result
$\lambda$	$f^{\prime\prime}\left( 0 \right)$	$f^{\prime\prime}\left( 0 \right)$	$f^{\prime\prime}\left( 0 \right)$
$0$	$1.0000$	$1.0000$	$1.0000$

**Table 3:** Effects of numerous parameters on Skin friction $({Cf}_{x})$.

$M$	${\epsilon }_{1}$	$\lambda$	$Fr$	$Q$	${Cf}_{x}$ ${\mathrm{ZnO}+SAE50}$	${Cf}_{x}$ $SAE50 (\phi =0)$
$0.1$	$0.1$	$0.4$	$0.3$	$0.02$	$0.8152$	$0.8593$
$0.2$					$0.8149$	$0.8539$
$0.3$					$0.8145$	$0.8521$
	$0.1$				$0.7167$	$0.7554$
$0.2$	$0.2$	$0.4$	$0.3$	$0.02$	$0.8172$	$0.8558$
	$0.3$				$0.9176$	$0.9562$
		$0.2$			$0.9056$	$0.9560$
$0.2$	$0.1$	$0.4$	$0.3$	$0.02$	$0.8045$	$0.8542$
		$0.6$			$0.7040$	$0.7499$
			$0.1$		$0.6126$	$0.6489$
$0.2$	$0.1$	$0.2$	$0.3$	$0.02$	$0.7131$	$0.7494$
			$0.5$		$0.8135$	$0.8498$
				$0.01$	$0.6995$	$0.7229$
$0.2$	$0.1$	$0.4$	$0.3$	$0.02$	$0.8076$	$0.8328$
				$0.03$	$0.9146$	$0.9415$

**Table 4:** Impacts of some parameters on Nusselt number ${(\mathrm{Nu}}_{x})$.

$S$	$Ec$	$Rd$	$Q$	${\epsilon }_{2}$	${\lambda }_{h}$	$\phi$	${\mathrm{Nu}}_{x}$(CWT)	${\mathrm{Nu}}_{x}$(NH)
$0.1$	$0.5$	$0.4$	$0.3$	$-0.1$	$0.1$	$0.0025$	$3.1778$	$1.9691$
$0.2$							$2.8573$	$1.5117$
$0.3$							$2.5812$	$1.2015$
	$0.1$						$3.1849$	$2.1707$
$0.2$	$0.2$	$0.3$	$0.1$	$-0.1$	$0.1$	$0.0025$	$3.1920$	$2.4579$
	$0.3$						$3.2016$	$2.7442$
		$0.1$					$3.1697$	$1.9606$
$0.2$	$2.0$	$0.3$	$0.1$	$-0.1$	$0.1$	$0.0025$	$3.1860$	$1.9777$
		$0.5$					$3.2023$	$1.9950$
			$0.2$				$2.3418$	$1.2273$
$0.2$	$2.0$	$0.3$	$0.4$	$-0.1$	$0.1$	$0.0025$	$1.9065$	$0.8948$
			$0.6$				$1.6072$	$0.6953$
				$-0.5$			$2.4453$	$1.2558$
$0.2$	$2.0$	$0.3$	$0.1$	$-0.3$	$0.1$	$0.0025$	$2.8148$	$1.5397$
				$-0.1$			$3.2023$	$1.9953$
					$0.2$		$3.1973$	$1.9749$
$0.2$	$2.0$	$0.3$	$0.1$	$-0.1$	$0.4$	$0.0025$	$3.1872$	$1.9343$
					$0.6$		$3.1770$	$1.8924$
						$0.0050$	$0.1555$	$0.0237$
$0.2$	$2.0$	$0.3$	$0.1$	$-0.1$	$0.7$	$0.0075$	$0.6675$	$0.1448$
						$0.0100$	$1.8150$	$0.7419$

**Table 5:** Effects of numerous parameters on Sherwood number $({Sh}_{x})$.

${\lambda }_{m}$	${\epsilon }_{3}$	$Rc$	$Sc$	$m$	${Sh}_{x}$ ${\mathrm{ZnO}+SAE50}$	${Sh}_{x}$ $SAE50 (\phi =0)$
$0.1$	$0.5$	$0.4$	$0.3$	$1.0$	$0.2576$	$0.2338$
$0.3$					$0.2583$	$0.2355$
$0.5$					$0.2591$	$0.2377$
	$0.1$				$0.1983$	$0.1742$
$0.3$	$0.3$	$0.4$	$0.3$	$1.0$	$0.2154$	$0.1921$
	$0.5$				$0.2325$	$0.2105$
		$0.2$			$0.2210$	$0.1997$
$0.3$	$0.5$	$0.4$	$0.3$	$1.0$	$0.2172$	$0.1959$
		$0.6$			$0.2135$	$0.1921$
			$0.1$		$0.2356$	$0.2146$
$0.3$	$0.5$	$0.4$	$0.3$	$1.0$	$0.2332$	$0.2122$
			$0.5$		$0.2307$	$0.2096$
				$1.0$	$0.2344$	$0.2135$
$0.3$	$0.5$	$0.4$	$0.3$	$1.5$	$0.2515$	$0.2304$
				$2.0$	$0.2772$	$0.2561$

## Conclusions

In present study, an attempt has been made to analyze heat and mass transfer characteristics of Darcy Forchheimer flow of magnetized nanolubricant ${\mathrm{ZnO}-SAE50}$ over Riga plate with Newtonian heating condition. Cattaneo–Christov model is used to assess heat and mass transmission rates. The evaluation of heat and mass transfer is additionally supported with heat radiation, uniform heat source/sink, viscous dissipation and higher order chemical reaction. Moreover, the influences of variable thermal conductivity, variable viscosity, variable diffusivity and the use of Patel model describe the novelty of our study. By introducing similarity quantities, the governing PDEs system is reformed to ODEs. The solution of the resulting system is then executed numerically by using MATLAB bvp-4c solver. The outcomes for various emerging parameters of interest are enlightened via tables and graphs. The followings are the major findings of this study:

- The growing values of porosity parameter, magnetic parameter, inertial parameter and variable viscosity parameter reduce the motion of the nanolubricant.
- The motion of the nanolubricant gets accelerated by enhancing the values of modified Hartmann number.
- The temperature of the nanolubricant goes up with the addition of more quantity of nanoparticles. Whereas, enhancing solid volume fraction causes a decline in the velocity profile.
- Heat absorption parameter and Prandtl number decrease temperature of the nanolubricant.
- The augmentation in the values of thermal radiation parameter, Eckert number, variable thermal conductivity parameter and heat generation parameter are helpful to enhance the temperature of the nanolubricant.
- The heat and mass transmission rates slow down for thermal and solutal time relaxation parameters respectively.
- The deterioration in concentration profile is caused due to growing values of chemical reaction parameter and Schmidt number.
- The concentration profile gets enriched by growing the order of the chemical reaction and variable mass diffusivity parameter.
- The value of Skin friction is less in the case of nanolubricant ${\mathrm{ZnO}-SAE50}$ as compared to $SAE50$ oil.
- In common wall temperature condition (CWT), greater thermal enhancement is achieved as compared to Newtonian heating (NH) case.
- The nanolubricant $\mathrm{ZnO}-SAE50$ offers better heat and mass transfer rates in comparison to $SAE50$ oil.

## Data Availability

All data generated or analyzed during this study is included in this article.

## List of symbols

$$u, v$$: Velocity components
${\mu }_{SAE50}$: Dynamic viscosity base fluid $(SAE50)$
${\mu }_{\mathrm{ZnO}-SAE50}$: Effective dynamic viscosity of nanolubricant $({\mathrm{ZnO}-SAE50})$
${\sigma }_{\mathrm{ZnO}}$: Electrical conductivity of ${\mathrm{ZnO}}$ np
${\sigma }_{SAE50}$: Electrical conductivity of base fluid $(SAE50)$
${\sigma }_{\mathrm{ZnO}-SAE50}$: Electrical conductivity of nanolubricant $(\mathrm{ZnO}-SAE50)$
$$T$$: Fluid temperature
${\rho }_{\mathrm{ZnO}-SAE50}$: Effective density of nanolubricant $(\mathrm{ZnO}-SAE50)$
${k}_{SAE50}$: Thermal conductivity of base fluid $(SAE50)$
${\rho }_{SAE50}$: Density of base fluid $(SAE50)$
${\rho }_{\mathrm{ZnO}}$: Density of np ${\mathrm{ZnO}}$
$\phi$: Solid volume fraction
$U$: External free stream velocity
${u}_{\mathrm{ZnO}}$: Brownian motion velocity of ${\mathrm{ZnO}}$ nps
$$c$$: Constant
$$Pe$$: Peclet number
${d}_{SAE50}$: Molecule size of $SAE50$
${d}_{\mathrm{ZnO}}$: Diameter of ${\mathrm{ZnO}}$ particle
${\alpha }_{SAE50}$: Thermal diffusivity of $SAE50$
${\in }_{1}$: Variable viscosity parameter
${\in }_{2}$: Variable thermal conductivity parameter
${\in }_{3}$: Variable diffusivity parameter
$$\beta$$: Width parameter
${D}_{\mathrm{ZnO}-SAE50}$: Diffusivity of nanolubricant $(\mathrm{ZnO}-SAE50)$
$m$: Order of chemical reaction
$Rc$: Chemical reaction parameters
$Q$: Modified Hartman number
$$\lambda$$: Porosity parameter
$$M$$: Magnetic parameter
$$Fr$$: Inertial parameter
${\lambda }_{h}$: Heat relaxation parameter
${\lambda }_{m}$: Mass relaxation parameter
$S$: Heat source parameter
${k}^{*}$: Permeability of porous medium
$F$: Non-uniform inertia coefficient
$${C}_{b}$$: Drag coefficient
$$Sc$$: Schmidt number
$$Rd$$: Radiation parameter
$$Pr$$: Prandtl number
$$Ec$$: Eckert number
${T}_{w}$: Temperature at the surface
$${T}_{\infty }$$: Ambient temperature
$$C$$: Fluid concentration
${C}_{w}$: Concentration at the surface
$${C}_{\infty }$$: Ambient concentration
$${D}_{SAE50}$$: Diffusivity of $$SAE50$$
${\left({C}_{p}\right)}_{SAE50}$: Specific HC of base fluid $(SAE50)$
${\left({C}_{p}\right)}_{ZnO}$: Specific HC of ${\mathrm{ZnO}}$ np
$\left({C}_{p}\right)\mathrm{ZnO}-SAE50$: Specific HC of nanolubricant $({\mathrm{ZnO}-SAE50})$
